# Targeting the Disease Response With NlpD and LytM for Effective Nonantibiotic Treatment of Urinary Tract Infections

**DOI:** 10.1093/infdis/jiag243

**Published:** 2026-05-06

**Authors:** Hien Thi Tran, Inês Gomes, Arunima Chaudhuri, Atefeh Nazari, Shahram Ahmadi, Hanna Végh, António N B M Carneiro, Urban Höglund, Christian Krintel, Catharina Svanborg, Ines Ambite

**Affiliations:** Department of Laboratory Medicine, Division of Microbiology, Immunology and Glycobiology, Lund University, Lund, Sweden; Department of Laboratory Medicine, Division of Microbiology, Immunology and Glycobiology, Lund University, Lund, Sweden; Department of Laboratory Medicine, Division of Microbiology, Immunology and Glycobiology, Lund University, Lund, Sweden; Department of Laboratory Medicine, Division of Microbiology, Immunology and Glycobiology, Lund University, Lund, Sweden; Department of Laboratory Medicine, Division of Microbiology, Immunology and Glycobiology, Lund University, Lund, Sweden; Department of Laboratory Medicine, Division of Microbiology, Immunology and Glycobiology, Lund University, Lund, Sweden; Department of Laboratory Medicine, Division of Microbiology, Immunology and Glycobiology, Lund University, Lund, Sweden; Legado Bio, Stockholm, Sweden; Department of Laboratory Medicine, Division of Microbiology, Immunology and Glycobiology, Lund University, Lund, Sweden; Department of Laboratory Medicine, Division of Microbiology, Immunology and Glycobiology, Lund University, Lund, Sweden; Department of Laboratory Medicine, Division of Microbiology, Immunology and Glycobiology, Lund University, Lund, Sweden

**Keywords:** nonantibiotic therapies, bacterial infections, acute pyelonephritis, acute cystitis, transcription inhibition

## Abstract

**Background:**

Finding new ways of treating bacterial infections is essential. The NlpD protein, which inhibits RNA polymerase II (Pol II), has shown therapeutic efficacy against urinary tract infection. This study investigated the mechanism of Pol II inhibition and protection by NlpD and its LytM peptide.

**Methods:**

Recombinant NlpD and LytM were screened for interactions with constituents of the Pol II complex, using AlphaFold predictions and protein interaction technology. Treatment effects were quantified in infected tissues and regulated host response pathways identified by genome-wide transcriptomics analysis in models of acute pyelonephritis and acute cystitis in *Irf3^−/−^* and *Asc^−/−^* mice, respectively.

**Results:**

LytM was shown to interact with constituents of the Pol II multiprotein complex, inhibiting the CDK12 kinase from phosphorylating the Pol II subunit RPB1 and disrupting Pol II complex formation by interfering with the interaction between PAF1C and RPB1. The protection by LytM against acute pyelonephritis was accompanied by a reduction in gene expression in infected kidneys from >1900 significantly regulated genes (fold change >6) in the placebo group to about 150 in LytM-treated mice. The inhibition of gene expression in infected kidneys particularly targeted the excessive innate immune response. A similar effect was observed in acute cystitis. Bacterial clearance was accelerated in both model by LytM treatment, with effects against antibiotic-sensitive and resistant *Escherichia coli* strains.

**Conclusions:**

The results suggest that inhibiting the disease response of the host, using NlpD or LytM, may offer an efficient alternative to antibiotics in these models.

## Teaser

NlpD and LytM are potential therapeutic alternatives to antibiotics, targeting the human RNA polymerase II multiprotein complex in infected tissues. By inhibiting the disease response to infection and accelerating bacterial clearance, they protect infected tissues with similar efficacy as antibiotics.

Extensive use of antibacterial drugs, particularly in common infections such as urinary tract infections (UTIs), correlates directly with the emergence of antibiotic resistance [1, 2], and the emergence of multiresistant bacterial strains has become a major global health concern [3, 4]. New therapeutic targets and disease susceptibility markers must therefore be identified and validated in clinical studies. The microbiome offers a rich source of beneficial molecules with a potential as novel therapeutics against infection [5–7]. Complex microbiomes have evolved molecular solutions for controlling the antibacterial defense of the hosts, molecules that may be used to develop alternative treatment concepts [8–10]. One example is the bacterial NlpD lipoprotein, which is highly conserved among *Escherichia coli* and other gram-negative bacteria [10] and part of an intricate enzymatic machinery that modifies the cell wall during bacterial cell division [11]. We have previously shown that the NlpD protein enters eucaryotic cells and targets and inhibits the RNA polymerase II (Pol II) phosphorylation machinery [9, 10]. The recombinant NlpD protein (rNlpD) has shown potent effects when used therapeutically in murine models of bacterial infection, inhibiting excessive innate immune responses and disease by attenuating gene expression in infected tissues [8, 10].

The NlpD protein was first identified in a loss-of-function mutant isolated from an asymptomatic human carrier of the asymptomatic bacteriuria strain *E coli* 83972. NlpD acts as an inhibitor of Pol II phosphorylation and a regulator of innate immune responses in human cells and murine infection models [9, 10]. This study investigated the molecular interactions of NlpD with the Pol II phosphorylation complex and the therapeutic potential of the NlpD protein and its LytM peptide in genetic models of acute cystitis and acute pyelonephritis, including infections caused by antibiotic-resistant bacteria. Treatment for 7 days with NlpD or LytM markedly reduced disease severity in infected kidneys and bladders and accelerated bacterial clearance in the urinary tract, including antibiotic-resistant strains. These potent protective effects are encouraging for continued studies of nonantibiotic treatment solutions for bacterial infections.

## MATERIALS AND METHODS

### In Silico Modeling

Target protein sequence were submitted to CoLabFold using AlphaFold2 multimer protocol [12] together with the LytM or NlpD sequence. For details, please see the Supplementary Material. A list of peptides used in this study is given in Supplementary Tables 3 and 4.

### Dot Blots and Western Blotting

Human kidney and lung epithelial cells were used. Cells were lysed using NP-40 lysis buffer or Qproteome Cell Compartment kit (Qiagen). For dot blots, 322 pmol recombinant proteins and 300 μg untreated whole cell lysate were used. Primary antibodies included anti-CDK12 (Abcam, catalog number [Cat#] ab57311), anti-Pol II (Santa Cruz Biotechnology, Cat# sc-899), anti-CDC73 (Biorbyt, Cat# orb101354), anti-NlpD [10], anti-β-actin (Sigma-Aldrich, Cat# A5441), and anti-histone H3 (abcam, Cat# ab1791) antibodies. For details, please see the Supplementary Material.

### Live Cell Imaging and Immunocytochemistry

Recombinant proteins were N-terminally labeled with AZ647 N-Hydroxysuccinimide ester. Cells were treated with recombinant proteins (6.5 µM) or 5,6-Dichloro-1-β-D-ribofuranosylbenzimidazole (DRB) (40–60 µM; Merck-Millipore, Cat# D1916) and stained with anti-phospho RPB1 (Merck-Millipore, Cat# 04-1571). Cells were imaged with a confocal microscope (LSM900, Carl Zeiss). For details, please see the Supplementary Material.

### Surface Plasmon Resonance Analysis

Real time biomolecular interaction analysis was performed by surface plasmon resonance (SPR) technology using Sensor Chip NTA (Cytiva Life Sciences, Cat# BR100034) on a Biacore X100 instrument, as previously described [13]. His-tagged NlpD (0.5 μM) and LytM (0.2–0.4 μM) were captured, followed by 5 different dilutions of peptides. For details, please see the Supplementary Material.

### Murine UTI Models

Female C57BL/6J *Asc*^−/−^, C57BL/6J *Irf3*^−/−^, or C57BL/6 [14] mice were treated with rNlpD protein or LytM by intraperitoneal (i.p.) injection. For toxicology studies, female NMRI mice were treated by intravenous injection. Mice were intravesically infected with *E coli* strain CY17 [15], CFT073 [16], or an extended-spectrum β-lactamase (ESBL)–producing clinical isolate [17], as previously described [17]. For outcome assessment and data analysis, investigators were blinded. For details, please see the Supplementary Material.

### Whole-Genome Transcriptome Analysis

Total RNA was extracted from murine kidneys as previously described [13]. Transcriptomics was done using Clariom S Mouse HT arrays (Thermo Fisher Scientific). Analysis was done using Ingenuity Pathway Analysis software (Qiagen). For details, please see the Supplementary Material.

### Statistical Analysis

Quantification Western blots and imaging was performed using Fiji software 1.46r (National Institutes of Health). For nonparametric data, Kruskal–Wallis test with post hoc Dunn multiple comparisons was used. Gaussian-distributed data, determined via the D’Agostino and Pearson normality test, was analyzed using 2-way ANOVA with post hoc Dunnett or Šídák multiple comparisons. Statistical significance was assessed using GraphPad Prism version 10 software, with significance assigned at *P* < .05.

### Study Approval

Experiments were approved by the Malmö/Lund Animal Experimental Ethics Committee at the Lund District Court, Sweden (#M119-16 and #6551-2021). Animal care and protocols followed institutional, national, and European Union guidelines and were governed by the European Parliament and Council Directive (2016/63, European Union), the Swedish Animal Welfare Act (Djurskyddslagen 1988:534), the Swedish Welfare Ordinance (Djurskydssförordningen 1988:539), and the Institutional Animal Care and Use Committee (IACUC) guidelines. The study was conducted in accordance with the Animal Research: Reporting of In Vivo Experiments (ARRIVE) guidelines (https://arriveguidelines.org).

## RESULTS

**Figure 1:**
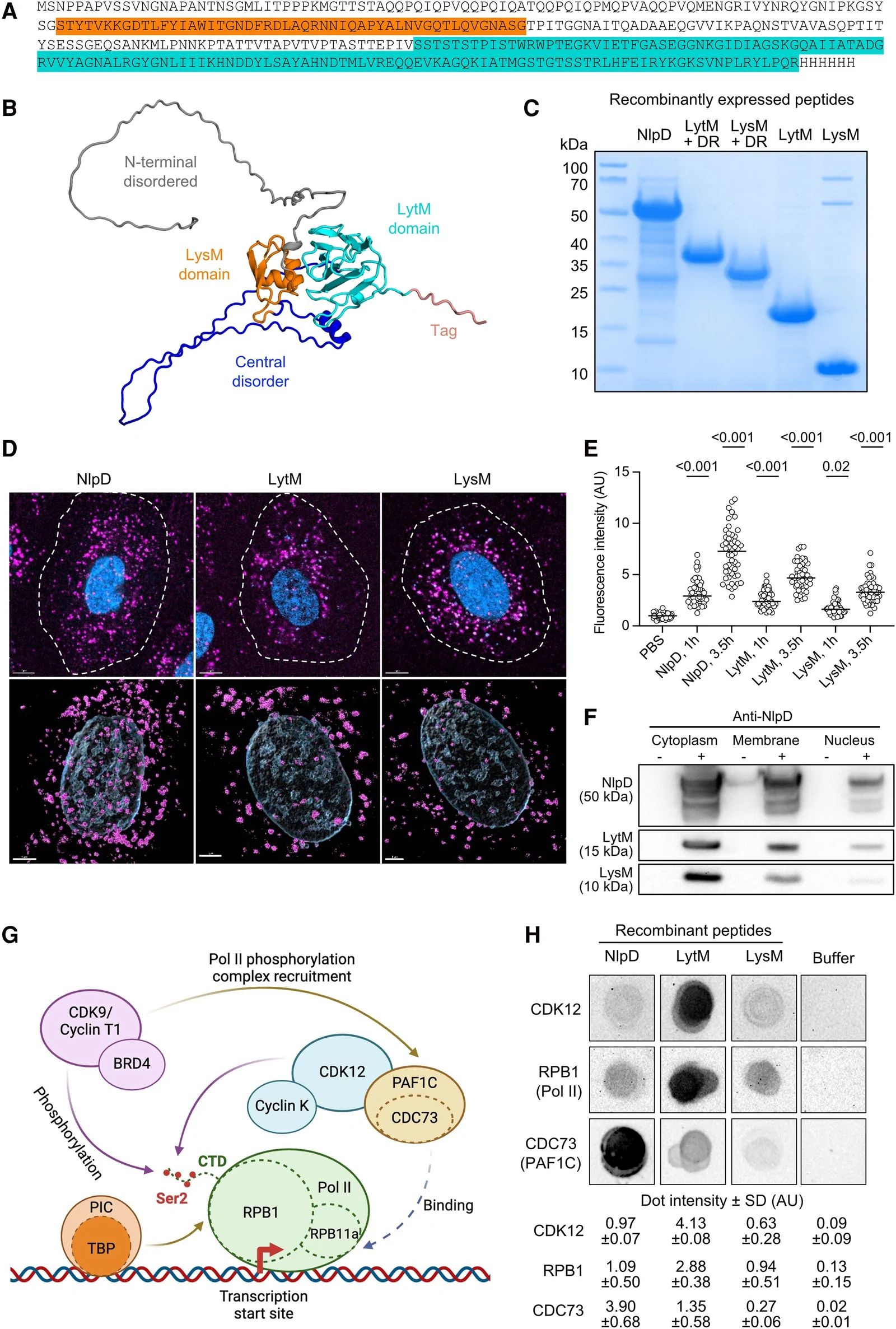
Structure and cellular interactions of the bacterial NlpD full protein and its globular peptide domains. *A*, Sequence of the recombinant NlpD (rNlpD) protein used in this study. The LysM (orange) and LytM (cyan) domains are highlighted. *B*, AlphaFold-generated model of rNlpD illustrating the close juxtaposition of the LysM (orange) and LytM (cyan) domains, surrounded by the N-terminal disordered (gray), the central disordered (blue), and the C-terminal purification 6× His tag (salmon). *C*, Purity of the recombinant proteins rNlpD, LytM, and LysM, verified by Coomassie staining. LytM with its N-terminal disordered region (LytM + DR) and LysM with its N-terminal disordered region (LysM + DR) were expressed as controls. *D*, Uptake of NlpD and the LytM and LysM peptides (6.5 μM) into the cytoplasm, and nuclei of human kidney cells (A498) visualized by live-cell confocal imaging of the AZ647-labeled peptides (magenta). Nuclei were counterstained with Hoechst (blue). Top panels: confocal images with dotted lines indicating cell periphery. Bottom panels: 3D reconstructions of the perinuclear and nuclear uptake in one representative cell. The nuclear surface is made transparent to visualize nuclear uptake. n = 2 experiments, scale bars = 3 μm. *E*, Quantification of time-dependent uptake of NlpD, LytM, and LysM (fluorescence intensity, 50 cells per sample, line represents the median). Kruskal–Wallis test followed by Dunn multiple comparisons test, compared to PBS control. *F*, Western blot of subcellular fractions from human lung cells (A549) cells exposed to rNlpD, LytM, or LysM (+), confirming the uptake of rNlpD, LytM, or LysM compared to untreated cells (−). *G*, Model of the Pol II phosphorylation complex at the transcription start site, indicating the CDK12 and cyclin K complex, the PAF1C complex including CDC73, as well as the Pol II complex including RPB1 and RPB11a. *H*, Dot blot analysis of NlpD, LytM, and LysM interactions with targets in whole cell lysates. Nitro-cellulose membranes primed with rNlpD, LysM, or LytM were overlaid with human kidney cell lysates, and binding of CDK12, RPB1, or CDC73 was detected using specific antibodies. Quantification of individual dot intensity is indicated, n = 2–3 experiments. Abbreviations: AU, arbitrary unit; PBS, phosphate-buffered saline; PIC, preinitiation complex; Pol II, RNA polymerase II; SD, standard deviation; TBP, TATA-binding protein.

To investigate the molecular basis of Pol II inhibition by NlpD and domains potentially interacting with the Pol II phosphorylation complex, a structure of rNlpD (amino acids [aa] 30–379, Figure 1*A*) was generated in silico, using AlphaFold (Figure 1*B*). The structure predicted a heterodimeric assembly of NlpD, with the globular LysM and LytM domains, flanked by 2 disordered regions. The prediction is consistent with the experimentally determined structures of LysM and LytM domain homologues [18, 19]. The NlpD protein and the LysM and LytM domains were recombinantly expressed and purified using a C-terminally positioned His-tag (>90% purity, Figure 1*C* and Supplementary Figure 1).

NlpD, LytM and LysM were internalized by human cells, as shown by live cell imaging using N-terminally AZ647-labeled peptides. Nuclear uptake was visualized by 3D z-stack reconstructions (Figure 1*D* and *E*). Uptake was confirmed by western blot analysis of subcellular fractions, with appropriate controls (Figure 1*F* and Supplementary Figure 2). Inhibition of Pol II phosphorylation was detected in cells exposed to LytM and NlpD, with a weaker effect of LysM. The effect of NlpD was comparable to the known chemical CDK9 inhibitor DRB [20] (Supplementary Figure 3).

An overview of the RNA Pol II phosphorylation complex is shown in Figure 1*G*. RPB1 polymerase activity is regulated through C-terminal phosphorylation by CDK9 and CDK12 [21–23]. The CDC73 protein constituent of the PAF1C complex supports transcriptional elongation and histone modifications by recruiting CDK12 to the RNA Pol II complex and CDK12 phosphorylates the RNA Pol II subunit RPB1 at Ser 2 [23–25], activating productive elongation of the nascent messenger RNA strand [26]. The NlpD protein and LytM and LysM peptides were shown to recognize CDK12, PAF1C, and RPB1, using a dot blot assay with human cell (Figure 1*H* and Supplementary Figure 1). LytM was shown to bind CDK12 and RPB1 more efficiently than NlpD, and binding to CDC73 was stronger for NlpD than LytM. LysM binding was weak.

### Interactions of NlpD, LytM, and LysM With CDK12, PAF1C, and RPB1, Investigated by In Silico Modeling and SPR

The AlphaFold Multimer protocol was used to predict domains in CDK12, RPB1, and PAF1C that interact with NlpD, LytM, and LysM. The resulting dimeric structures underwent visual inspection using PyMOL and were ranked according to their interface predicted template modelling (ipTM) scores.

**Figure 2.**
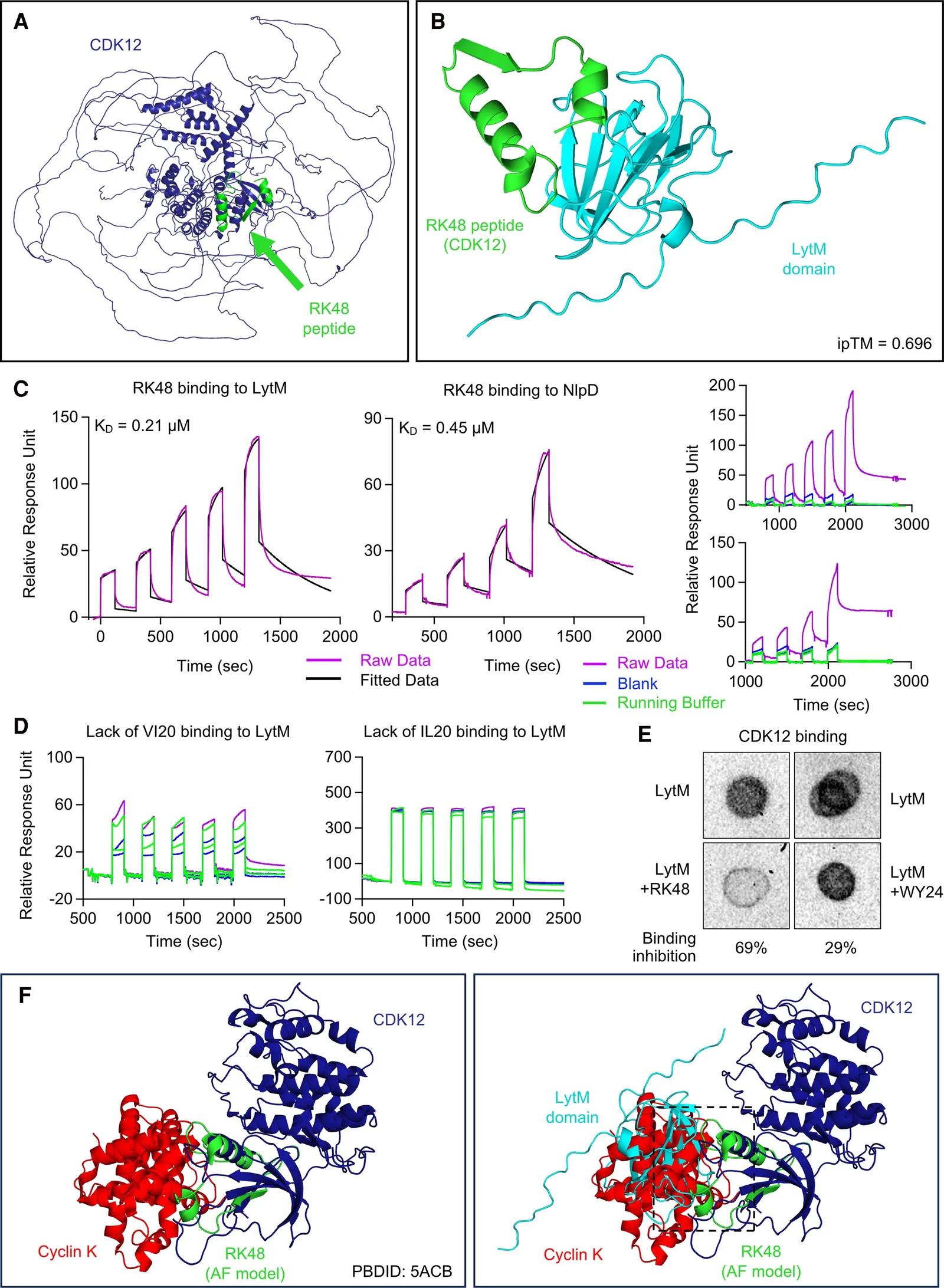
Predicted interactions between CDK12 and LytM. *A*, AlphaFold-generated structure of human CDK12 with indicated LytM binding site, in the CDK12 kinase domain, shown in green (aa 759–806, RK48 peptide). *B*, Predicted interaction between LytM (cyan) and the RK48 peptide (green) in the kinase domain of CDK12. *C*, Surface plasmon resonance binding analysis showing the dose-dependent interaction of LytM and NlpD with the RK48 peptide. The His-tagged recombinant LytM and NlpD proteins were captured on Sensor Chip NTA and flooded with increasing concentrations of the RK48 peptide (magenta). Runs with assay running buffer (green) and experimental blank (blue). Blank-subtracted data were fitted to a 1:1 Langmuir binding model (black). Equilibrium K_D_ of bindings are indicated. *D*, Lack of binding of shorter RK48 peptides. *E*, Inhibition of LytM binding to CDK12 by RK48. Pretreatment of LytM with RK48 in solution inhibited the subsequent binding to CDK12 in the dot blot assay. WY24 peptide (aa 719–742) does not inhibit LytM binding. *F*, Overlay of the human CDK12:cyclin K complex crystal structure (PDB 5ACB) with the modeled CDK12:LytM complex, predicted by the AlphaFold multimer protocol. For clarity, only the CDK12 region interacting with LytM (RK48, green) is shown, together with LytM (cyan), CDK12 (blue), and cyclin K (red). Abbreviations: K_D_, equilibrium dissociation constant; ipTM, interface predicted template modelling; AF, AlphaFold.

The globular kinase domain of CDK12 (aa 701–1100) was submitted with the LytM sequence to the AlphaFold Multimer protocol. CDK12 residues 758–806 were predicted to interact with LytM (RK48, ipTM = 0.696; Figure 2*A* and *B* and Supplementary Figure 4) and with the rNlpD protein (ipTM = 0.408) but not with LysM alone (ipTM = 0.156–0.252, Supplementary Table 1). The interactions were confirmed by SPR analysis (Figure 2*C* and Supplementary Figure 5). The RK48 peptide bound to LytM and NlpD, in a dose-dependent manner (equilibrium dissociation constant [K_D_] = 0.21 μM and 0.45 μM, respectively), but smaller peptide fragments of RK48 were not recognized (Figure 2*D*). Binding of LytM to RK48 was further verified by competitive inhibition using the dot blot assay (Figure 2*E*). As the RK48 sequence is positioned at the cyclin K binding site of CDK12 [27] the interaction was predicted to inhibit the kinase activity of CDK12 and subsequent RPB1 phosphorylation (Figure 2*F* and Supplementary Figure 6).

**Figure 3.**
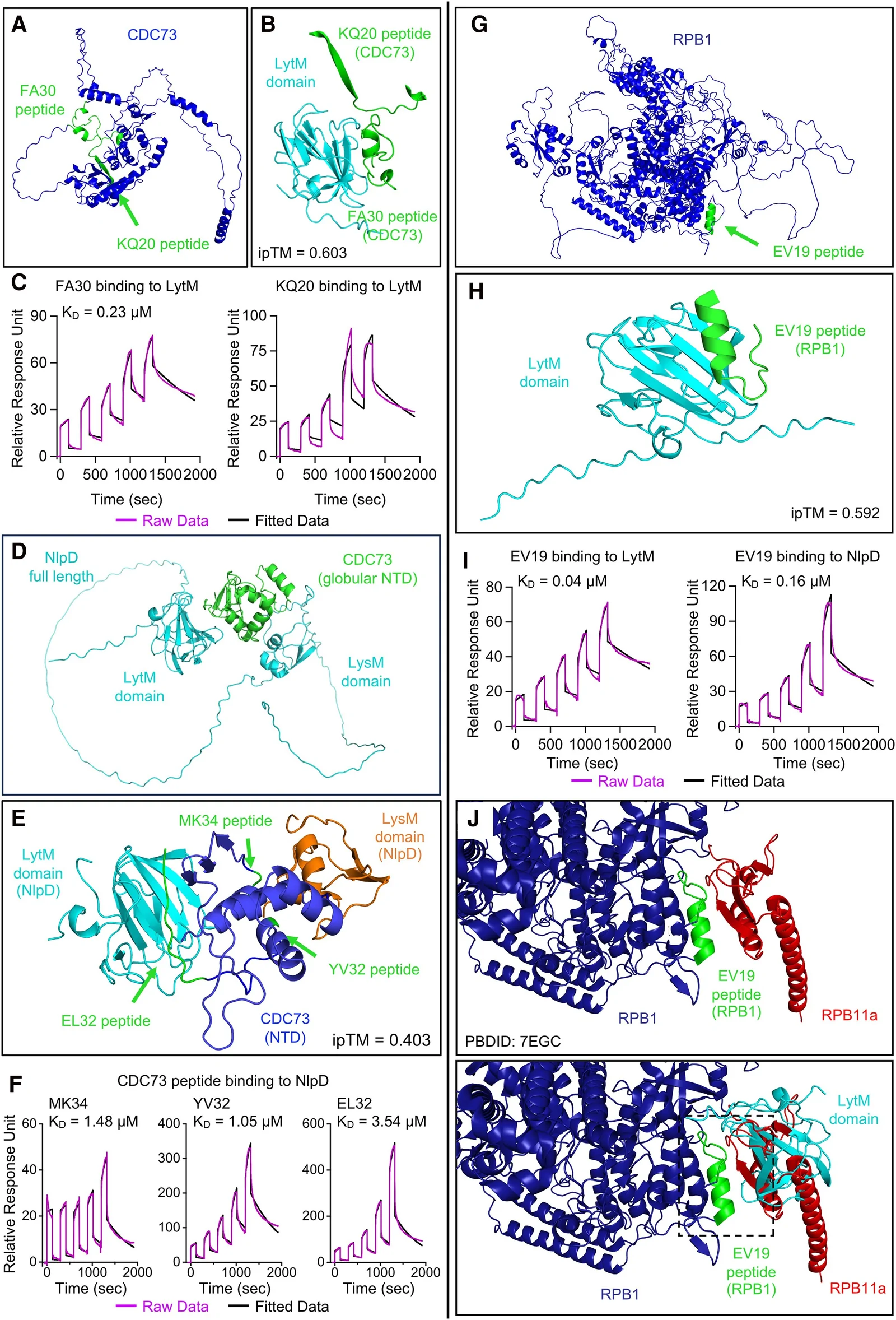
Predicted interactions between CDC73 and LytM, and RPB1 and LytM. *A*, AlphaFold-generated structure of human CDC73 with indicated LytM binding sites, shown in green (aa 298–327, FA30 peptide; aa 392–411, KQ20). *B*, Predicted interaction between LytM (cyan) and the KQ20 and FA30 peptides of CDC73 (green). *C*, SPR binding analysis showing the dose-dependent interaction of LytM with the FA30 and KQ20 peptides. The His-tagged recombinant LytM was captured on Sensor Chip NTA and flooded with increasing concentrations of the FA30 and KQ20 peptides (magenta). Blank-subtracted data were fitted to a 1:1 Langmuir binding model (black). *D*, Predicted interaction between NlpD (cyan) and the CDC73 globular N-terminal domain (green). *E*, AlphaFold-generated structure of the globular N-terminal domain of CDC73 (blue) with indicated NlpD binding sites, shown in green (aa 1–34, MK34 peptide; aa 54–85, YV32; aa 101–132, EL32). Predicted interaction between the LytM domain (cyan) with EL32 peptide, and the LysM domain (orange) with MK34 and YV32 peptides of CDC73 (green). *F*, SPR binding analysis showing the dose-dependent interaction of NlpD with the MK34, YV32, and EL32 peptides. The His-tagged recombinant NlpD was captured on Sensor Chip NTA and flooded with increasing concentrations of the MK34, YV32, and EL32 peptides (magenta). Blank-subtracted data were fitted to a 1:1 Langmuir binding model (black). *G*, AlphaFold-generated structure of RPB1 with indicated LytM binding site, shown in green (aa 556–574, EV19 peptide). *H*, Predicted interaction between LytM (cyan) and the EV19 peptide of RPB1 (green). *I*, SPR binding analysis showing the dose-dependent interaction of LytM and NlpD with the EV19 peptide. The His-tagged recombinant LytM and NlpD proteins were captured on Sensor Chip NTA and flooded with increasing concentrations of the EV19 peptide (magenta). Blank-subtracted data were fitted to a 1:1 Langmuir binding model (black). Equilibrium K_D_ of bindings are indicated. *J*, Overlay of the human RPB1:RPB11a complex (PDB 7EGC) with the modeled RPB1:LytM complex, predicted by AlphaFold multimer protocol. For clarity, only the RPB1 region interacting with LytM (EV19, green) is shown, together with, LytM (cyan), RPB1 (blue), and RPB11a (red). Abbreviations: K_D_, dissociation constant; ipTM, interface predicted template modelling; NTD, N-terminal domain; SPR, surface plasmon resonance.

The CDC73 sequence from PAF1C was further probed for interactions with LytM and LysM, (Figure 3*A*). CDC73 residues 303–321 and 400–403, corresponding to the semi-disordered central region and C-terminal globular domain of CDC73, were predicted to interact with LytM (ipTM = 0.603) and binding was confirmed by SPR analysis, using peptides FA30 (aa 298–327) and KQ20 (aa 392–411) (Figure 3*B* and *C*, K_D_ = 0.2 3μM) and validated in vitro by dot blots (Supplementary Figure 7). LysM alone was not predicted to bind CDC73 (ipTM = 0.106–0.301; Supplementary Table 1).

The full-length rNlpD was predicted to interact with CDC73, through its LytM and LysM domains (ipTM = 0.403; Figure 3*D* and *E*). SPR analysis confirmed binding of rNlpD to 3 CDC73 peptides, aa 1–34 (MK34), aa 54–85 (YV32), and aa 101–132 (EL32) at micrometer concentrations (Figure 3*F* and Supplementary Figure 5). The results suggest that LytM and rNlpD target several regions of CDC73 predicting that their binding would impair the structural flexibility required for the formation of a functional PAF1C complex and its interaction with RPB1 (Supplementary Figure 7).

Segments of RPB1 were probed for interactions with LytM (Supplementary Figure 8), and residues 556–574 (EV19, ipTM = 0.592) in the globular domain were predicted as targets (Figure 3*G* and *H*). Binding of LytM to EV19 was confirmed by SPR analysis (K_D_ = 0.04 μM, Figure 3*I* and Supplementary Figure 5). rNlpD was also predicted to bind to this region of RPB1 (residues 531–801, ipTM = 0.356) and shown to interact with EV19 (K_D_ = 0.16 μM), supporting this RPB1 segment as the preferred binding site.

To evaluate the functional consequences of these interactions, electron microscopy (EM) structures of the full RNA Pol II complex were reviewed [28–33]. Notably, residues 556–574 form the interface of RPB1 with RPB11a, in the preinitiation complex, as demonstrated by several EM studies and recently by cryo-EM of the integrator-containing PP2A-AC paused elongation complex (Supplementary Figure 9). The interaction between RPB1 and RPB11a across all experimental structures supports the importance of this interaction for the Pol II complex [28–33]. The predicted interaction with the EV19 peptide therefore suggests that LytM would interfere with the formation of an active complex containing RPB1 and RPB11a, presumably attenuating the transcriptional process (Figure 3*J*). Colocalization of NlpD with the identified targets was observed in cell nuclei and confirmed by line scans (Supplementary Figure 10).

### Biodistribution and Tolerability of NlpD and LytM

**Figure 4.**
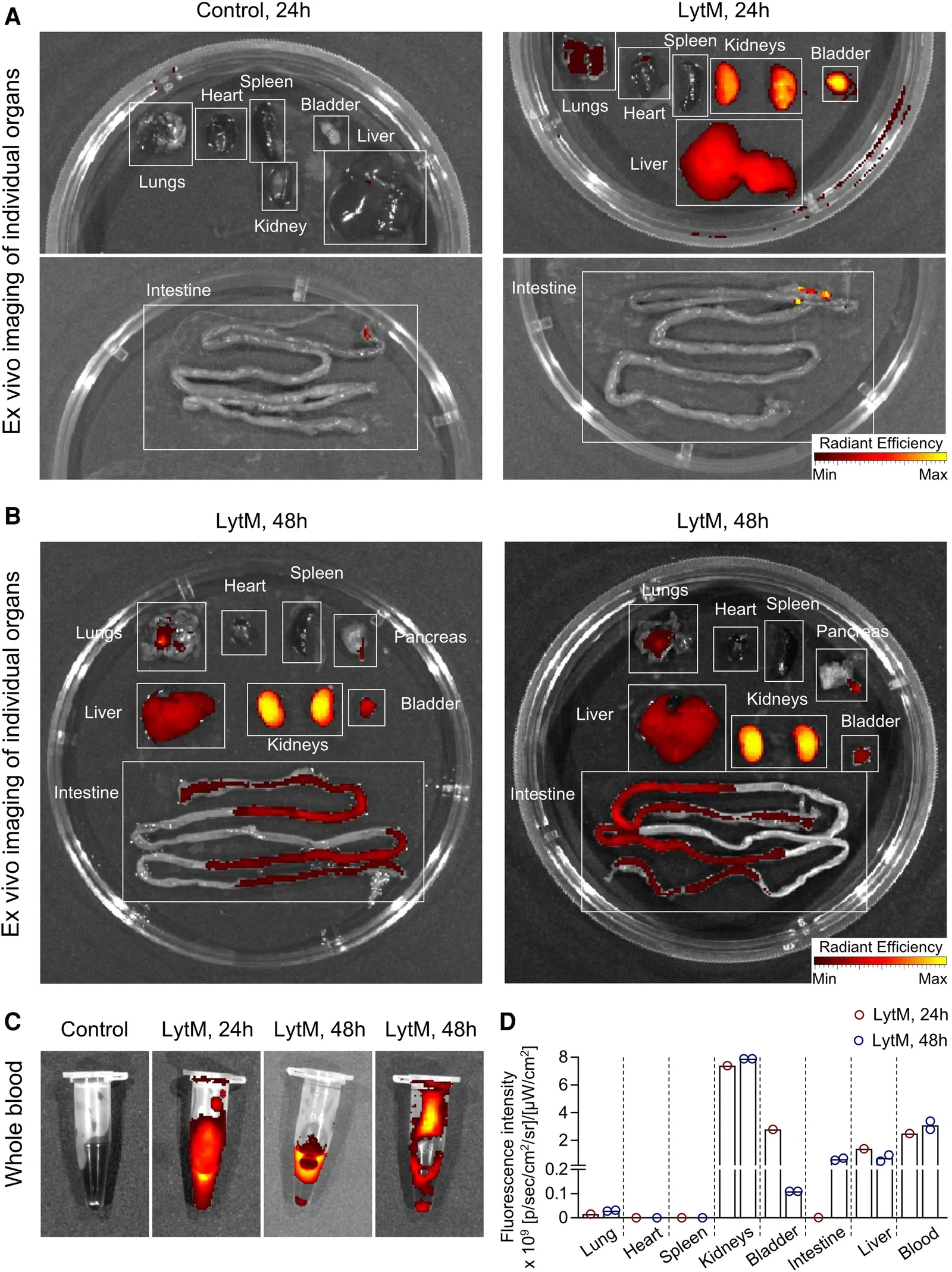
Biodistribution of LytM in treated mice. In vivo fluorescence imaging (using In Vivo Imaging System [IVIS]) of LytM biodistribution after intraperitoneal injection. C57BL/6 mice were administered AZDye 647–labeled LytM (42.6 μg, 0.36 mg/mL), 24 and 48 hours prior to IVIS imaging. *A*, Ex vivo images of organs from controls and mice receiving LytM taken 24 hours after injection. *B*, Ex vivo images of organs from mice receiving LytM taken 48 hours after injection. *C*, Fluorescence signaling imaging from blood samples. *D*, Quantification of fluorescence intensity using regions of interest in IVIS software. Data show individual mice and median.

The biodistribution of LytM was investigated in vivo by whole body fluorescent imaging (In Vivo Imaging System [IVIS]) technology, after intraperitoneal injection of C57BL/6 mice with the AZDye 647–labeled peptide (Figure 4). A strong fluorescence signal was detected in kidneys, bladders, and blood samples from treated mice, 24 and 48 hours postinjection. A less intense fluorescence signal was also detected in the livers and intestines of treated mice.

The tolerability of NlpD was tested in NMRI mice treated by daily intravenous administration for 7 days and compared to phosphate-buffered saline (PBS). Animals were monitored for changes in body weight, routine hematological and clinical chemistry markers, organ weights, and health status, during administration and for 7 days (Supplementary Figure 11 and Supplementary Table 2). NlpD treatment was associated with a minimal decrease in body weight compared to PBS controls and slight local irritation during treatment, which had subsided at recovery. NlpD was otherwise well tolerated with no associated changes in hematological or clinical chemistry parameters, nor any macroscopic pathologies in excised organs (Supplementary Table 2). There was no evidence of cytotoxicity in NlpD- or LytM-treated human cells, defined by Adenosine TriPhosphate quantification and PrestoBlue staining (Supplementary Figure 12).

The results suggest that NlpD and LytM have affinity for the urinary tract organs and are well tolerated by treated animals.

### Therapeutic Effects of LytM in Murine Infection Models Compared to rNlpD

**Figure 5.**
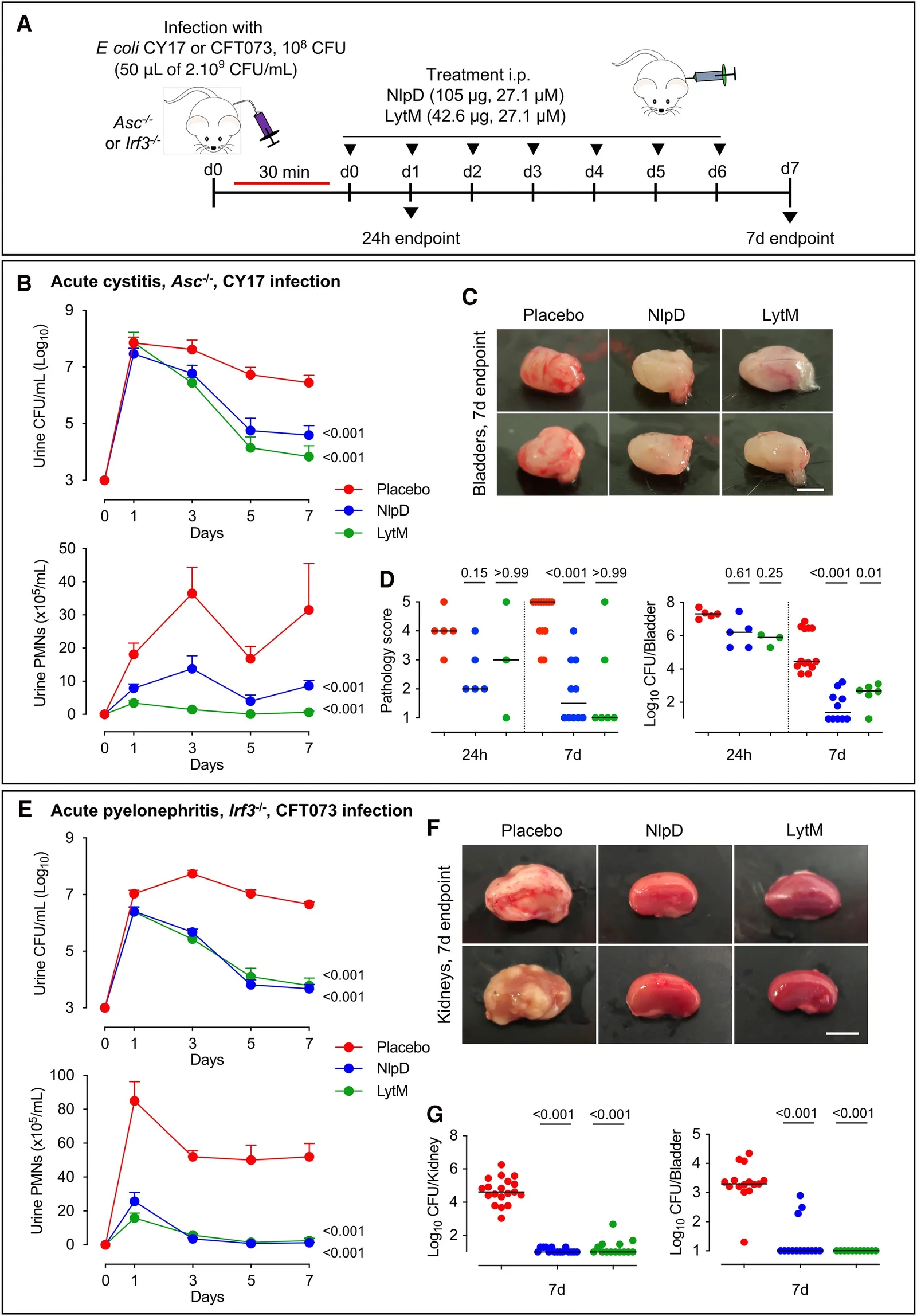
Protective effect of rNlpD and LytM treatment in a murine acute infection model. *A*, Schematic of the protocol used for rNlpD and LytM treatments of *Escherichia coli* infection in genetically susceptible mice. Mice were administered rNlpD (100 μL, 27.1 μM, 105 μg) or LytM (100 μL, 27.1 μM, 42.6 μg) i.p. once a day for 7 days starting at the time of intravesical infection with *E coli* (10^8^ CFU). Placebo control mice received i.p. injections of PBS (100 μL). *B*, *Asc^−/−^* mice were infected with *E coli* CY17 and infection was monitored for 7 days. Lower bacterial count in urine and urine neutrophil count in mice treated with rNlpD (blue) or LytM (green) compared to placebo (red). n = 2–3 independent experiments for each treatment, for a total of 18 (PBS), 15 (NlpD), and 6 (LytM) mice; data are presented as mean ± SEM and analyzed using 2-way ANOVA with Šídák multiple comparisons test compared to the placebo group. *C*, Example of macroscopic appearance of bladder from *Asc^−/−^* mice in the placebo and the rNlpD and LytM treatment groups, 7 days after infection. Scale bar = 2 mm. *D*, Lower bladder gross pathology and bacterial count in bladder tissues in treated mice compared to placebo. Individual animals are shown, lines represent medians and data were analyzed using Kruskal–Wallis followed by Dunn multiple comparisons test compared to the placebo group. *E*, *Irf3^−/−^* mice were infected with *E coli* CFT073 and infection was monitored for 7 days. Lower bacterial count in urine and urine neutrophil count in mice treated with rNlpD (blue) or LytM (green) compared to placebo (red). n = 3 independent experiments for each treatment, for a total of 24 (PBS), 19 (NlpD), and 14 (LytM) mice; data are presented as mean ± SEM and analyzed using 2-way ANOVA with Dunnett multiple comparisons test compared to the placebo group. *F*, Example of macroscopic appearance of kidneys from *Irf3^−/−^* mice in the placebo and rNlpD and LytM treatment groups, 7 days after infection. Scale bar = 5 mm. *G*, Lower bacterial count in kidney and bladder tissues in treated mice compared to placebo, 7 days after infection. Lines represent medians and data were analyzed using Kruskal–Wallis followed by Dunn multiple comparisons test compared to the placebo group. Abbreviations: CFU, colony-forming units; i.p., intraperitoneal; PBS, phosphate-buffered saline; PMNs, polymorphonuclear leukocytes; SEM, standard error of the mean.

Using relevant disease models is critically important to evaluate if new treatments are sufficiently potent to treat severe infections. The therapeutic potential of LytM was examined in 2 bacterial infection models; acute cystitis, where disease is limited to the urinary bladder, and acute pyelonephritis, where one or both kidneys are affected (Figure 5). Susceptibility to acute cystitis is increased in *Asc*^−/−^ mice, which develop an IL-1β overexpression disorder and severe bladder pathology within 7 days of *E coli* infection [34]. Susceptibility to acute pyelonephritis is increased in *Irf3^−/−^* mice, which develop a MYC and IRF7 overexpression disorder and severe kidney pathology within 7 days of infection, due to a loss of transcriptional control of innate immunity [35]. A schematic of the infection and treatment protocols is shown in Figure 5*A*.

*Asc* ^−/−^ mice were infected by intravesical injection of the acute cystitis strain *E coli* CY17 and sacrificed after 24 hours or 7 days (Figure 5*B*–*D*). Disease severity was defined macroscopically, by gross pathology scores, defined by enlarged and hyperemic, inflamed bladders, high bacterial counts in urine and bladder tissues, and elevated neutrophil counts in urine. *Irf3*^−/−^ mice were infected by intravesical injection of *E coli* CFT073 and sacrificed after 7 days (Figure 5*E*–*G*). Gross pathology was defined by enlarged, hyperemic kidneys; abscess formation; and disease severity marked by elevated histopathology scores, high bacterial counts in urine and renal tissues, and elevated urine neutrophil counts.

LytM was administered i.p. (27.1 µM, 100 µL), 30 minutes after infection and daily for 7 days. rNlpD-treated mice were included as positive controls and the placebo group received PBS. The severity of disease was markedly reduced by LytM treatment in the acute pyelonephritis and acute cystitis models, compared to placebo. Abscess formation and renal hyperemia were inhibited, and bacterial and neutrophil counts in urine and renal tissue were reduced.

### Attenuated Gene Expression Profiles in Infected Kidneys After LytM and NlpD Treatment

**Figure 6.**
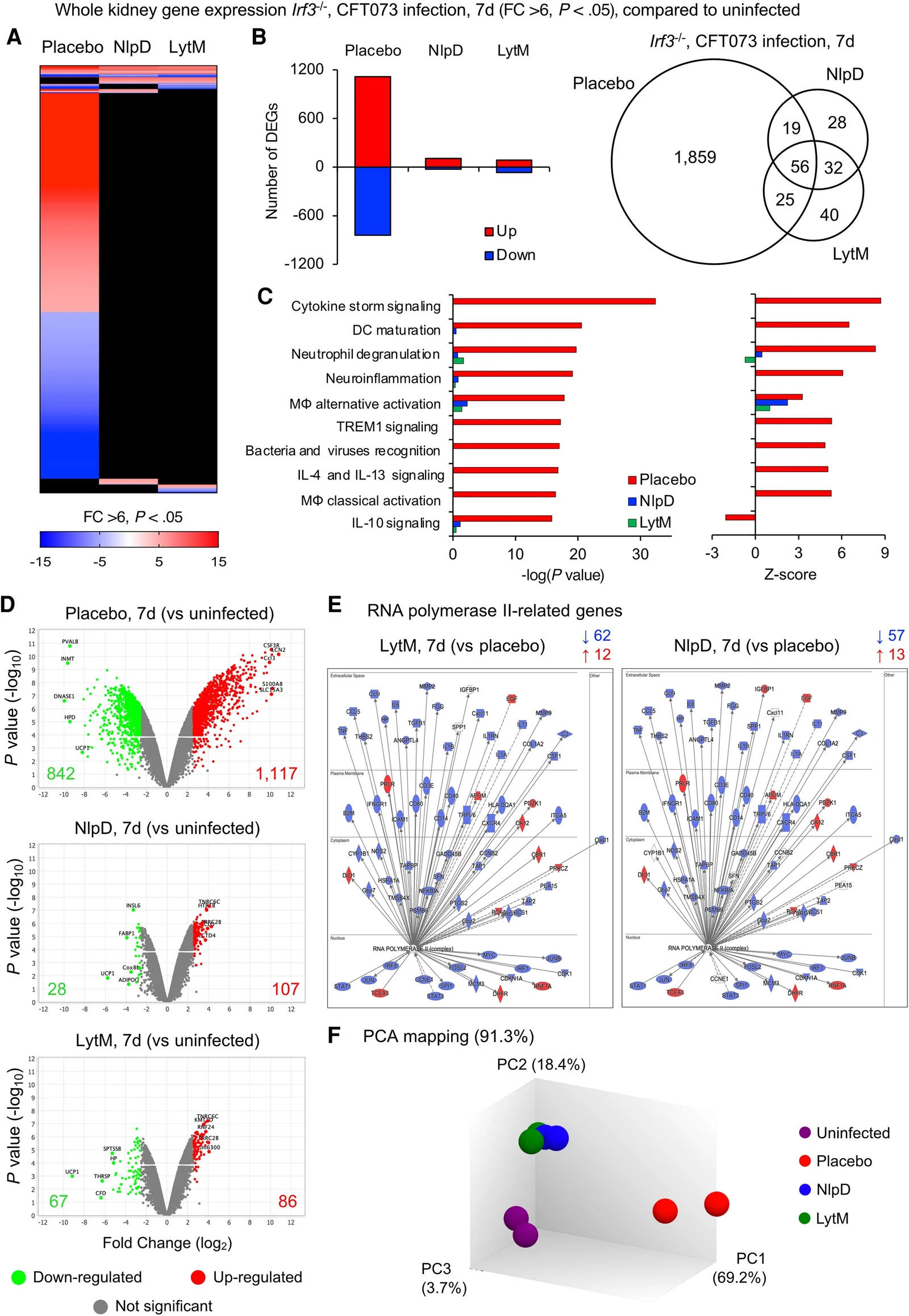
Protective inhibition of the disease response defined by gene expression analysis. *A*, Total renal RNA was isolated from infected mice or uninfected mice after 7 days and was subjected to genome-wide transcriptomic analysis. Disease-associated genes were identified by comparing the infected mice to uninfected controls (heatmap, cutoff FC >6, *P* < .05). Response to kidney infection in the placebo group, showing total regulated genes (n = 2 per group; red, upregulated; blue, downregulated; cutoff FC >6, *P* < .05 compared to uninfected mice). Lack of disease response in mice treated with NlpD or LytM. *B*, Inhibition by rNlpD or LytM treatment of response to infection, shown as a marked reduction in the number of DEGs in treated mice compared to the placebo group. *C*, Functional analysis of expressed genes, identifying a strong activation of an innate immune response (cytokine storm signaling, neutrophil degranulation, neuroinflammation, macrophage activation, bacterial recognition) in the placebo group and a lack of response in the rNlpD- or LytM-treated groups. The activation Z score for each pathway is shown. Data normalized against healthy, uninfected mice. *D*, Volcano plots of all the detected genes (n = 19 863 genes) identifying up-regulated (red) and down-regulated (green) genes in the treatment and placebo groups (n = 2 per group, 7 days postinfection compared to uninfected; gray, no change; red, activated; green, inhibited). *E*, Network analysis of the RNA polymerase II–related genes regulated by rNlpD and LytM treatment in the infected mice. RNA polymerase II–related genes were inhibited compared to the placebo group (n = 2 per group, 7 days postinfection compared to placebo; white, no change; red, activated; blue, inhibited). *F*, PCA of the whole kidney RNA data sets. The left cluster shows uninfected mice (purple), and the adjacent cluster shows the rNlpD- and LytM-treated mice (blue and green). The right cluster shows the placebo group (red), distant from the other samples. Abbreviations: DC, dendritic cell; DEG, differentially expressed gene; FC, fold change; IL, interleukin; MΦ, macrophage; PCA, principal component analysis.

Gene expression was strongly attenuated in the kidneys of LytM-treated *Irf3^−/−^* mice, compared to placebo (Figure 6, Supplementary Table 3, and Supplementary Figure 13). A high cutoff (fold change [FC] 6, *P* < .05), was selected to focus the analysis on the most strongly regulated genes. Using this cutoff, there were 1959 genes regulated by infection in the placebo group compared to 153 genes in the LytM-treated group (Figure 6*B*) and 135 genes in NlpD-treated mice.

Specifically, LytM treatment virtually abolished the overactivation of innate immunity that accompanies disease in this model (Figure 6*C* and *D*), including the cytokine storm response, and the activation of neutrophil degranulation and macrophage-related pathways. LytM was further shown to specifically inhibit an RNA Pol II–dependent gene network, including several transcriptional regulators (Figure 6*E*).

A similar effect on gene expression was detected in the kidneys of NlpD-treated mice. Principal component analysis of the whole RNA data set clustered LytM- or NlpD-treated *Irf3^−/−^* mice together, adjacent to uninfected mice, while the placebo group clustered at a distance (69% variance, first principal component, Figure 6*F*).

### Treatment of Antibiotic-Resistant *E coli*

**Figure 7.**
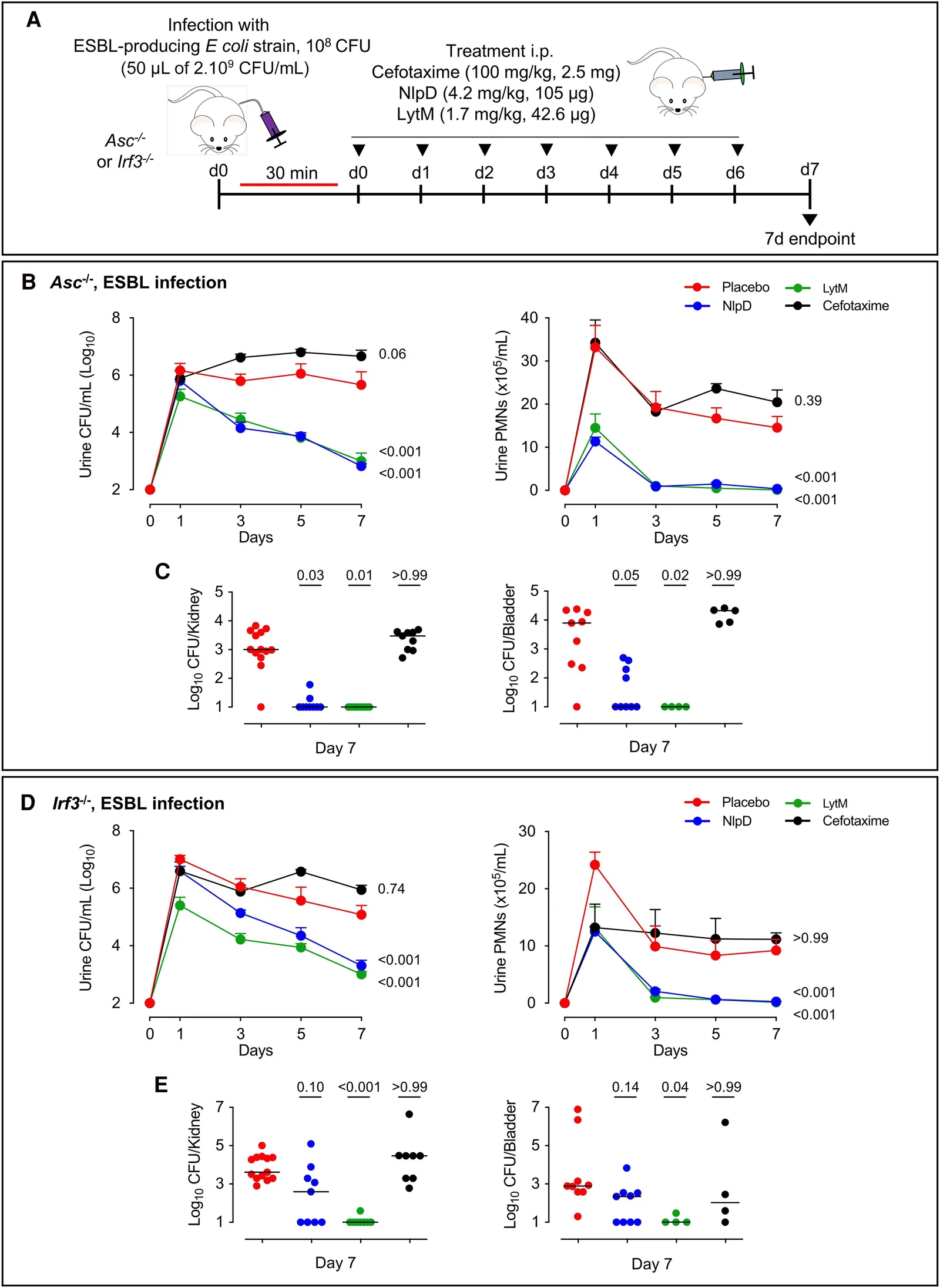
Efficacy of rNlpD and LytM treatment in mice infected with antibiotic-resistant *Escherichia coli*. *A*, Schematic of the protocol used for treatment of infection caused by an ESBL-producing *E coli* strain (10^8^ CFU) in *Asc^−/−^* and *Irf3^−/−^* mice. Mice were administered rNlpD (100 μL, 4.2 mg/kg, 105 μg), LytM (100 μL, 27.1 μM, 42.6 μg), or cefotaxime antibiotic (100 μL, 100 mg/kg, 2.5 mg) i.p. once a day for 7 days starting at the time of intravesical infection. Placebo control mice received i.p. injections of PBS (100 μL). *B*, *Asc^−/−^* mice were infected with an ESBL-producing *E coli* and infection was monitored for 7 days. Lower bacterial count in urine and bladder tissue and urine neutrophil count in mice treated with rNlpD (blue) or LytM (green) compared to placebo (red). Cefotaxime treatment group (black) did not differ from the placebo group. n = 2 independent experiments for each treatment, for a total of 13 (PBS), 9 (NlpD), 8 (LytM), and 9 (cefotaxime) mice; data are presented as mean ± SEM and analyzed using 2-way ANOVA with Šídák multiple comparisons test compared to the placebo group. *C*, Lower bacterial count in bladder tissues in treated mice compared to placebo. Lines represent medians and data were analyzed using Kruskal–Wallis followed by Dunn multiple comparisons test compared to the placebo group. *D*, *Irf3^−/−^* mice were infected with an ESBL-producing *E coli* and infection was monitored for 7 days. Lower bacterial count in urine and urine neutrophil count in mice treated with rNlpD (blue) or LytM (green) compared to placebo (red), 7 days after infection. The cefotaxime treatment group (black) did not differ from the placebo group. n = 2 independent experiments for each treatment, for a total of 13 (PBS), 9 (NlpD), 8 (LytM), and 9 (cefotaxime) mice; data are presented as mean ± SEM and analyzed using 2-way ANOVA with Šídák multiple comparisons test compared to the placebo group. *E*, Lower bacterial count in kidney tissues in treated mice compared to placebo. Lines represent medians and data were analyzed using Kruskal–Wallis followed by Dunn multiple comparisons test compared to the placebo group. Abbreviations: CFU, colony-forming units; ESBL, extended-spectrum β-lactamase; i.p., intraperitoneal; PBS, phosphate-buffered saline; PMNs, polymorphonuclear leukocytes; SEM, standard error of the mean.

There were no direct antibacterial effects of NlpD or LytM on uropathogenic *E coli* strains, quantified as growth curves in broth (Supplementary Figure 14). To investigate treatment effects against infections caused by antibiotic-resistant strains [17], *Asc*^−/−^ and *Irf3*^−/−^ mice were infected with an ESBL-producing *E coli* isolate from the urinary tract (Figure 7). NlpD or LytM treatment was administered i.p. 30 minutes after infection and daily for 7 days (Figure 7*A*). The placebo group received PBS. Cefotaxime treatment was administered following the same protocol (100 mg/kg, 100 µL i.p.) and treatment effects were quantified after 7 days.

The *Asc*^−/−^ mice developed acute cystitis and the *Irf3*^−/−^ mice developed acute pyelonephritis, defined by gross pathology, neutrophil recruitment, and bacterial counts, both with reduced disease severity compared to *E coli* CY17 and CFT073 infections. The rNlpD- and LytM-treated mice showed a marked reduction in acute cystitis severity (Figure 7*B* and *C*) and in acute pyelonephritis severity (Figure 7*D* and *E*) compared to the placebo group. Neutrophil recruitment was inhibited and bacterial clearance accelerated in rNlpD- and LytM-treated mice compared to placebo. In contrast, there was no effect of cefotaxime treatment on the disease end points or bacterial clearance, compared to placebo.

The potent inhibition of disease by LytM and NlpD treatment confirms the importance of the interactions predicted in silico and by SPR analysis and the resulting lack of activation of the Pol II machinery and the response to infection.

## DISCUSSION

This study investigated the treatment effects of the NlpD protein and its LytM peptide in models of UTI. The results show that these molecules, which lack antibiotic activity, act by targeting the disease response in the host, particularly the innate immune response during the first week of infection. By attenuating this response, disease severity is reduced and bacterial clearance accelerated, with similar kinetics as antibiotics. The NlpD protein and LytM peptide enter host cells, target the RNA polymerase machinery, and inhibit Pol II phosphorylation. This study investigated the molecular basis of these effects, showing that the globular LytM domain of NlpD interferes with the phosphorylation of the Pol II subunit RPB1, by direct interaction with RPB1, by inhibiting the CDK12 kinase that phosphorylates RPB1, and by the assembly of PAF1C and RPB1 into a functional Pol II complex. NlpD and LytM were both shown to have affinity for the urinary tract organs after i.p. injection and were well tolerated by treated animals. The findings provide a molecular context for the nonantibiotic treatment effects of LytM and NlpD in the UTI models.

The results suggest that NlpD and LytM are more selective and less toxic than Pol II inhibitors previously investigated, such as DRB [36]. The development of Pol II inhibitors has mainly focused on small molecules for cancer treatment, linked to their ability to induce cellular apoptosis [37]. Most Pol II inhibitors target cyclin-dependent kinases (CDKs), primarily CDK9, and have major toxicity [37–39]. Unlike these inhibitors, NlpD did not affect CDK9 or cell viability [10], suggesting that by inhibiting later steps in the Pol II cycle, NlpD achieves a greater level of precision and lower toxicity. Furthermore, the specificity of NlpD for the Pol II complex may be increased by targeting PAF1C, which is essential for the recruitment of CDK12 to RPB1 [25]. Based on the binding affinities predicted by SPR, the interactions of NlpD and LytM with their cellular targets would be predicted to create transient and reversible PoI II inhibition, in contrast to small chemical inhibitors like DRB, which are irreversible and have strong cytotoxic effects [36].

The Pol II cycle involves sequential steps that need to be activated to produce functional RNA transcripts [40]. By inhibiting the phosphorylation of RPB1 and RNA elongation, NlpD would be expected to broadly inhibit transcription, with general consequences for diverse cellular functions. The effects of NlpD and LytM treatment were surprisingly specific for infected tissues, as disease-associated genes were strongly inhibited but not homeostatic gene expression. Furthermore, LytM and NlpD were well tolerated in healthy animals [10], where toxicity studies did not detect significant effects of NlpD after intravenous administration. Cell viability was maintained, in vitro, in cells exposed to NlpD or LytM. The potent treatment effect and regained innate immune balance may explain why bacterial clearance is accelerated in the treated mice. A restored, more balanced innate immune response may help clear the infection by conventional means, such as neutrophils and antibacterial peptides. Resolving the mechanisms of disease specificity and bacterial clearance are interesting future challenges.

The results illustrate the importance of treating disease caused by infection and not just the infection. Genetic susceptibility factors have been identified in the murine model of UTI, and different transcriptional regulators have been shown to control disease development in acute pyelonephritis and acute cystitis. Overactivation of innate immunity and complex cytokine storm patterns characterizes acute pyelonephritis in *Irf3^−/−^* mice, and overactivation of IL-1 and inflammasome pathways characterizes acute cystitis in *Asc^−/−^* mice [34, 35]. These disease phenotypes resemble human disease, and findings in the murine models have been validated in human genetic studies [41–43]. Acute pyelonephritis is frequently accompanied by urosepsis, a major cause of mortality worldwide, and sepsis-related disease end points have been observed in the murine UTI model [44]. Smith et al studied the response to intravenous infection with *E coli* CFT073 in a murine model and characterized bacterial virulence factors associated with bacterial persistence [45]. The infection models used here will be of interest for future analyses of urosepsis and host determinants of disease severity. In view of the potent treatment effects of NlpD and LytM in acute pyelonephritis, which includes infections caused by antibiotic-resistant *E coli* strains, their effects should be further explored.

## Supplementary Material

jiag243_Supplementary_Data

## Supplementary Data

Supplementary materials are available at *The Journal of Infectious Diseases* online (http://jid.oxfordjournals.org/). Supplementary materials consist of data provided by the author that are published to benefit the reader. The posted materials are not copyedited. The contents of all supplementary data are the sole responsibility of the authors. Questions or messages regarding errors should be addressed to the author.
