# Supplementary material for: Targeting the Disease Response With NlpD and LytM for Effective Nonantibiotic Treatment of Urinary Tract Infections
Source: J Infect Dis. 2026 May 6;234(2):e316–29. doi: 10.1093/infdis/jiag243 (PMC13537167; doi:10.1093/infdis/jiag243)
Supplement: Supplementary file 1 [file jiag243_supplementary_data.pdf]

Supplementary Figure 1

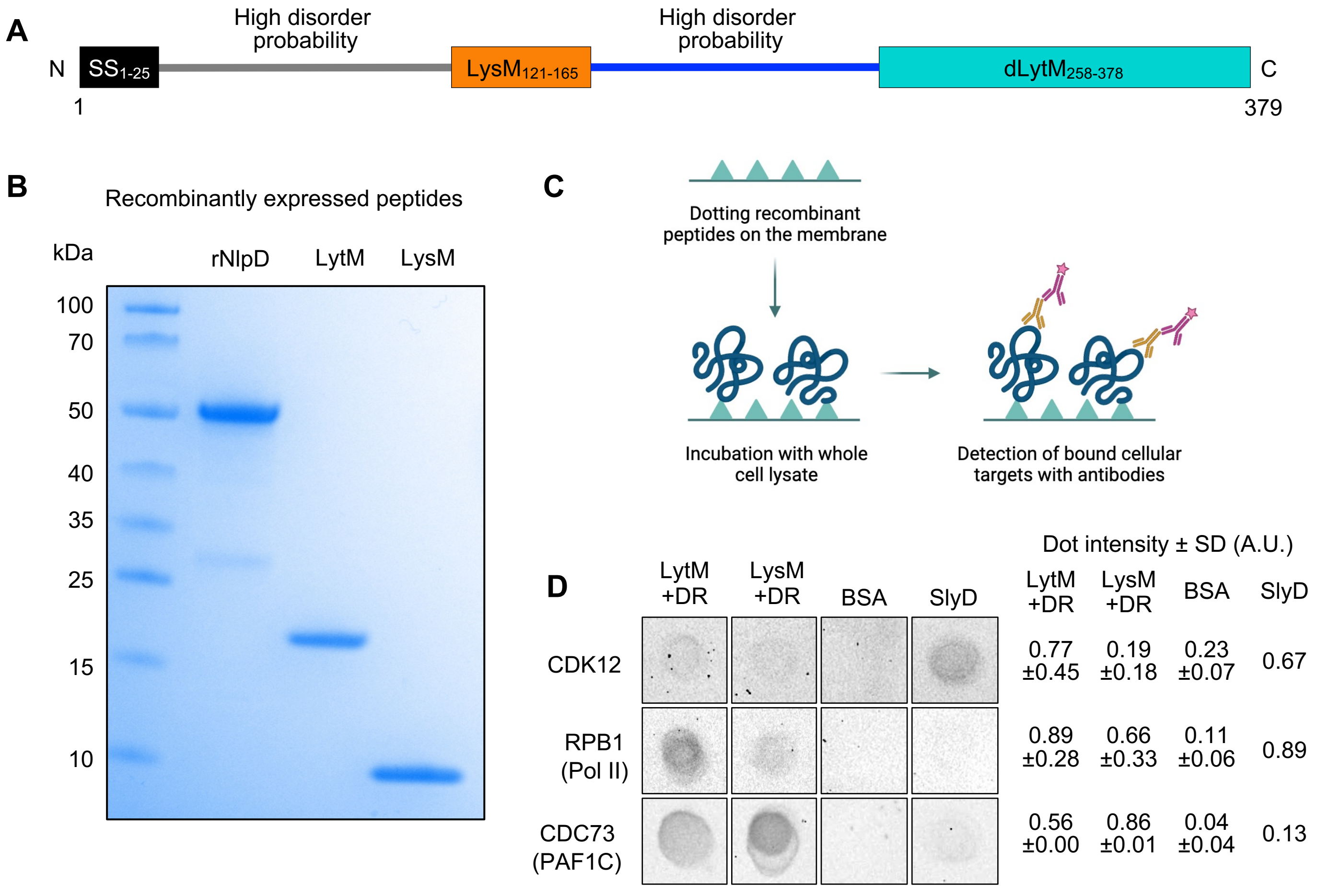

Supplementary Figure 1. Controls for dot blot analysis of interactions with cellular targets. (**A**) Domain organization of the *E. coli* NlpD protein (40 kDa, UniProt P0ADA3): 25 amino acid (aa) signal peptide, N-terminal disordered region (95 aa, grey), LysM domain (45 aa, orange), central disordered region (93 aa, blue), degenerate LytM (dLytM) domain (120 aa, cyan). (**B**) Purity of the recombinant proteins rNlpD, LytM and LysM, verified by Coomassie staining. (**C**) Schematics of the dot blot method used in Fig. 1E. (**D**) Controls for the CDK12, CDC73 and RPB1 dot blot analysis. LytM and LysM with their respective N-terminal disordered region (LytM+DR and LysM+DR), bovine serum albumin (BSA) and an unrelated bacterial protein (SlyD) were used. Quantification of individual dot intensity are indicated.

Supplementary Figure 2

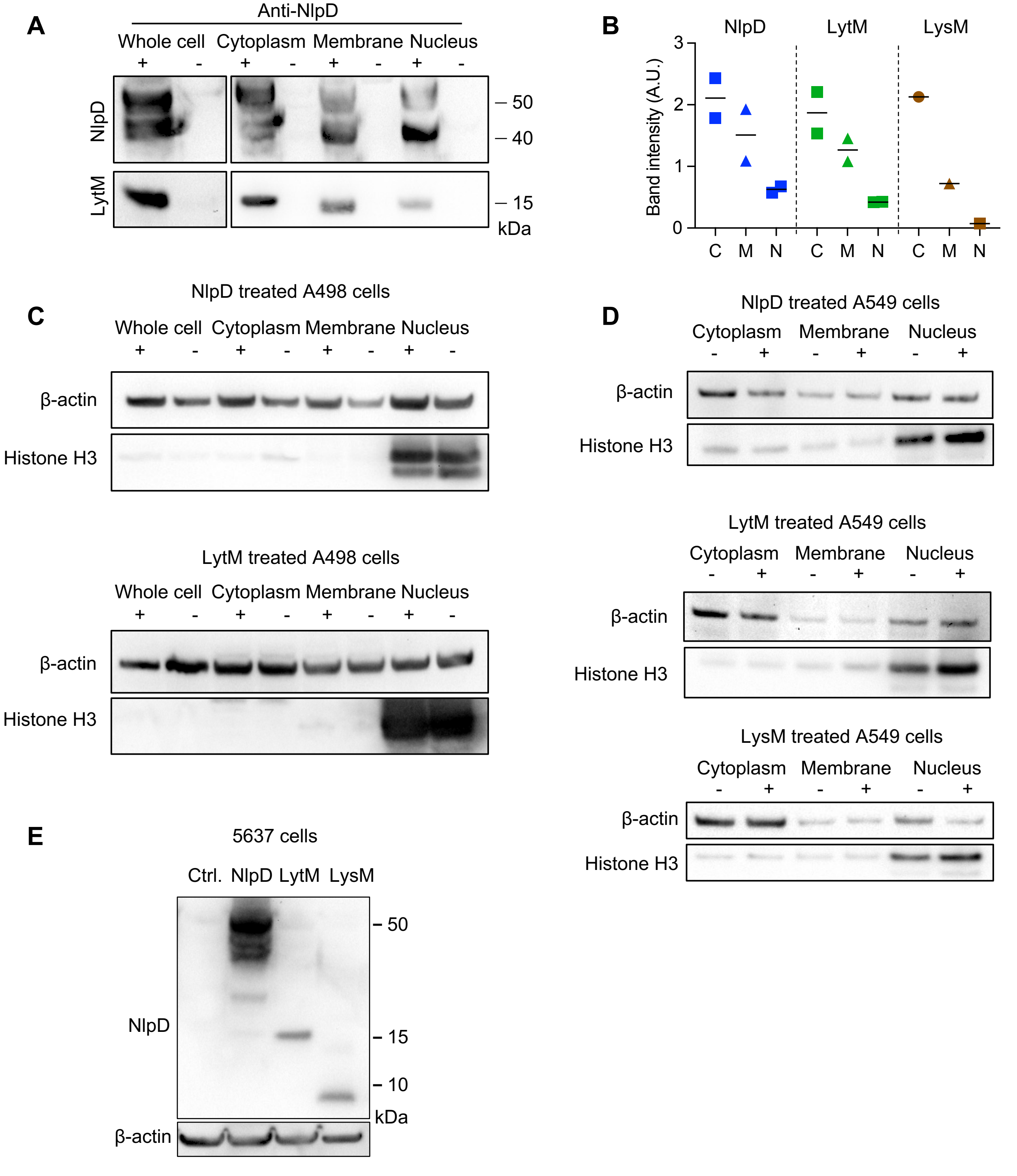

Supplementary Figure 2. Controls for Western blot of cellular uptake of rNlpD, LytM and LysM. (**A**) Western blot of rNlpD and LytM (+) in whole cell extracts and sub-cellular fractions from treated A498 cells, compared to untreated cells (-), *n* = 2 experiments. LysM was not detected by Western blot in treated A498 cells. (**B**) Quantification of NlpD, LytM and LysM uptake in different sub-cellular fractions detected by Western blot experiments. (**C**) β-actin and histone H3 controls for Western blot of whole cell extracts and sub-cellular fractions from A498 cells. (**D**) β-actin and histone H3 controls for Western blot of whole cell extracts and sub-cellular fractions from A549 cells. (**E**) Western blot of rNlpD, LytM and LysM in whole cell extracts from treated 5637 cells.

Supplementary Figure 3

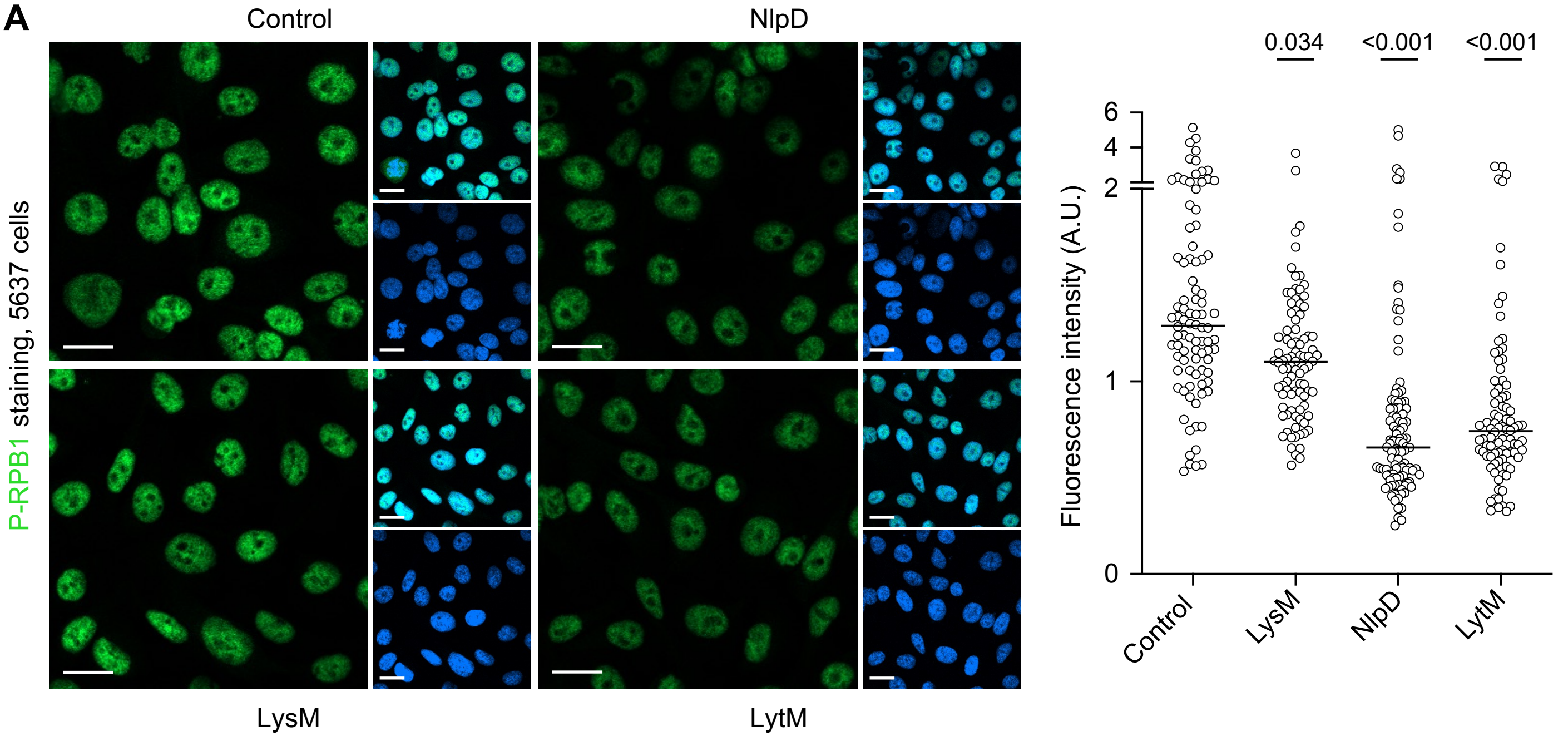

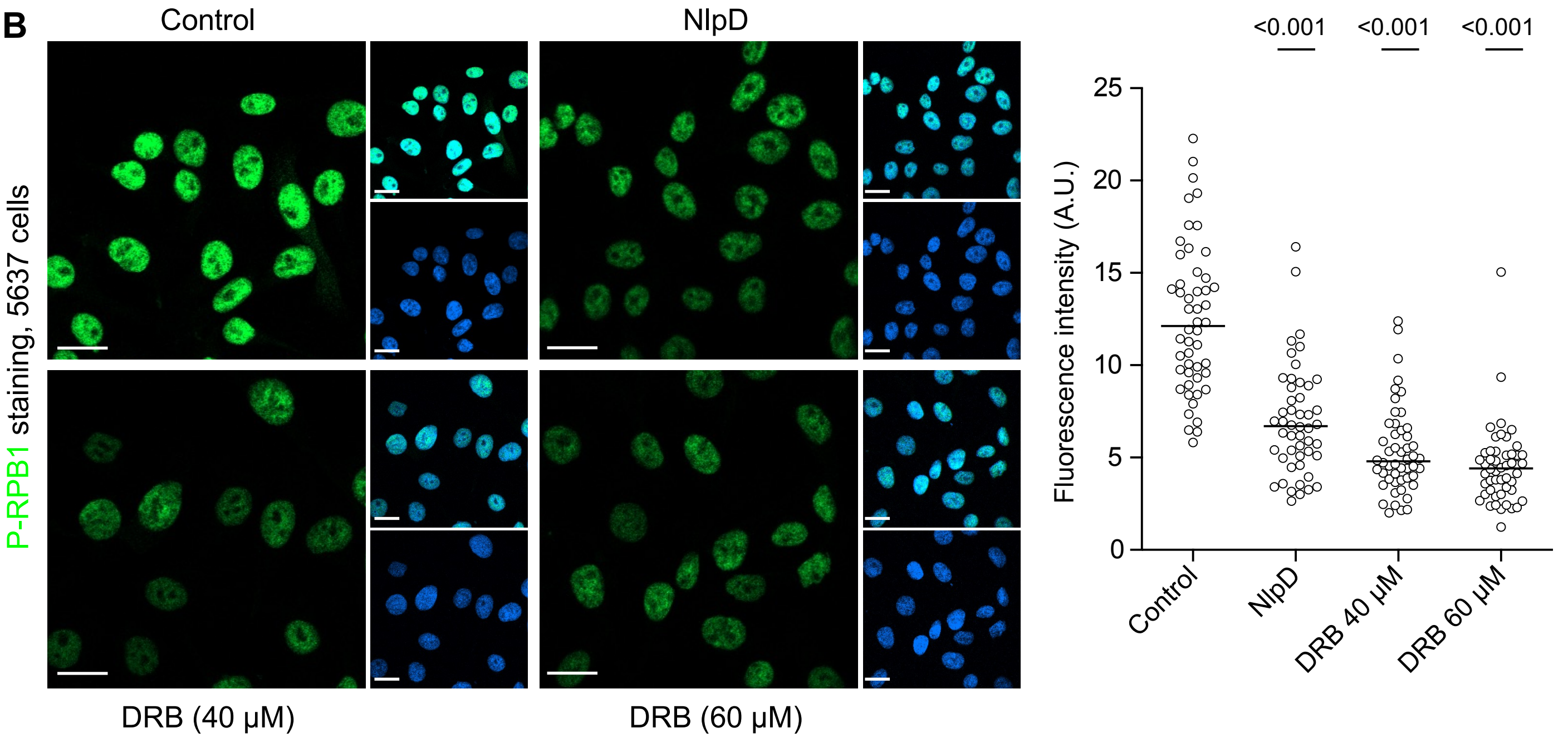

Supplementary Figure 3. Inhibition of Pol II phosphorylation by NlpD, LytM and LysM, compared to DRB.

(**A**) Staining of Serine 2 phosphorylated-RPB1 (green) in human bladder cells (5637) treated with rNlpD, LytM or LysM peptides (6.5 μM, 3h) compared to untreated cells. Pol II phosphorylation was strongly inhibited by NlpD and LytM and weakly inhibited by LysM treatment, compared to untreated controls. Quantification of fluorescence intensity, *n* = 90 cells per sample, bars represent medians, analyzed using Kruskal-Wallis followed by Dunn's multiple comparison test compared to PBS control. (**B**) Staining of Serine 2 phosphorylated-RPB1 (green) in 5637 cells treated with rNlpD (6.5 μM, 3h) compared to cells treated with DRB, a chemical Pol II inhibitor. Quantification of fluorescence intensity, *n* = 50 cells per sample, bars represent medians, analyzed using Kruskal-Wallis followed by Dunn's multiple comparison test compared to PBS control. Nuclei were counterstained with Hoechst (blue). Scale bars 20 μm.

Supplementary Figure 4

A Scoring for LytM interaction with CDK12

Target region (residues)	pTM	ipTM	Rank (region)	Rank (protein)
**Disordered 1 1-393**	0.311	0.279	1	10
	0.309	0.230	2	16
	0.306	0.199	3	17
	0.302	0.190	5	19
	0.312	0.198	4	18
**Disordered 2 380-740**	0.334	0.385	3	6
	0.318	0.134	5	20
	0.322	0.350	4	7
	0.332	0.463	1	4
	0.339	0.424	2	5
**Kinase domain 701-1100**	0.663	0.299	5	9
	**0.750**	**0.696**	**1**	**1**
	0.668	0.342	4	8
	0.725	0.523	3	3
	0.686	0.559	2	2
**Disordered 3 1091-1490**	0.334	0.238	5	15
	0.332	0.246	3	13
	0.340	0.276	1	11
	0.331	0.244	4	14
	0.339	0.256	2	12

Kinase domain

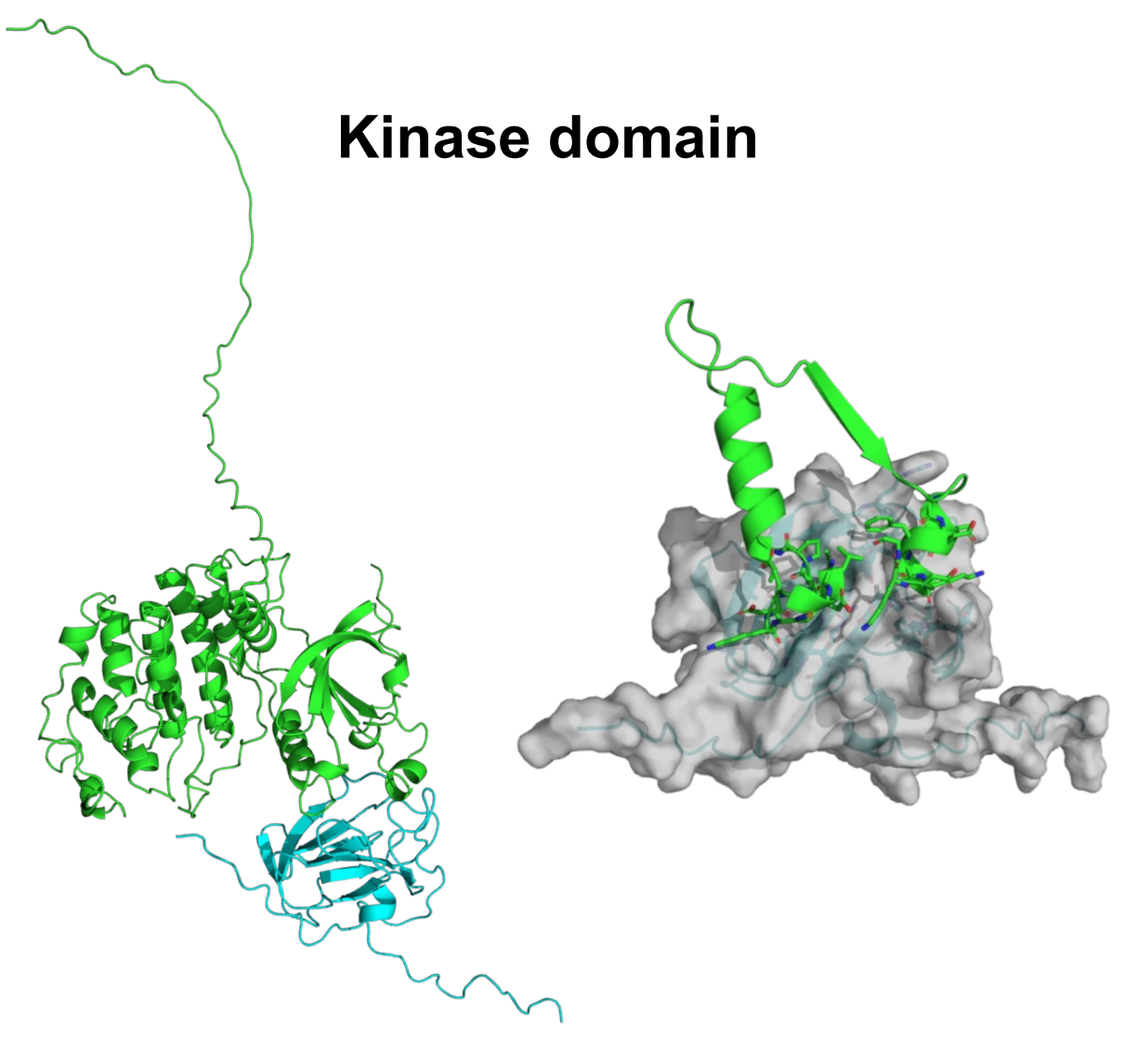

B Top ranked LytM interaction with CDK12

Disordered 1

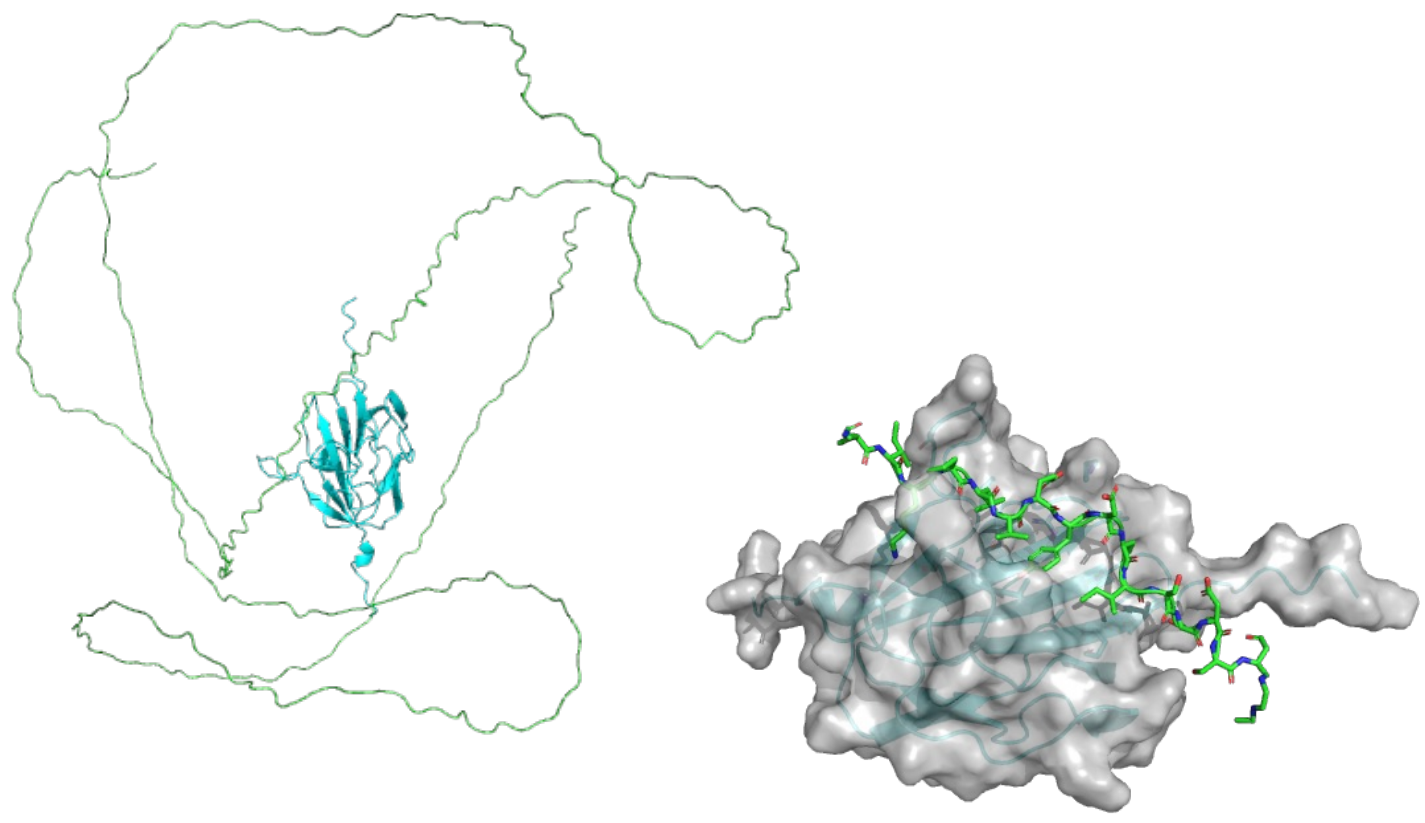

Disordered 2

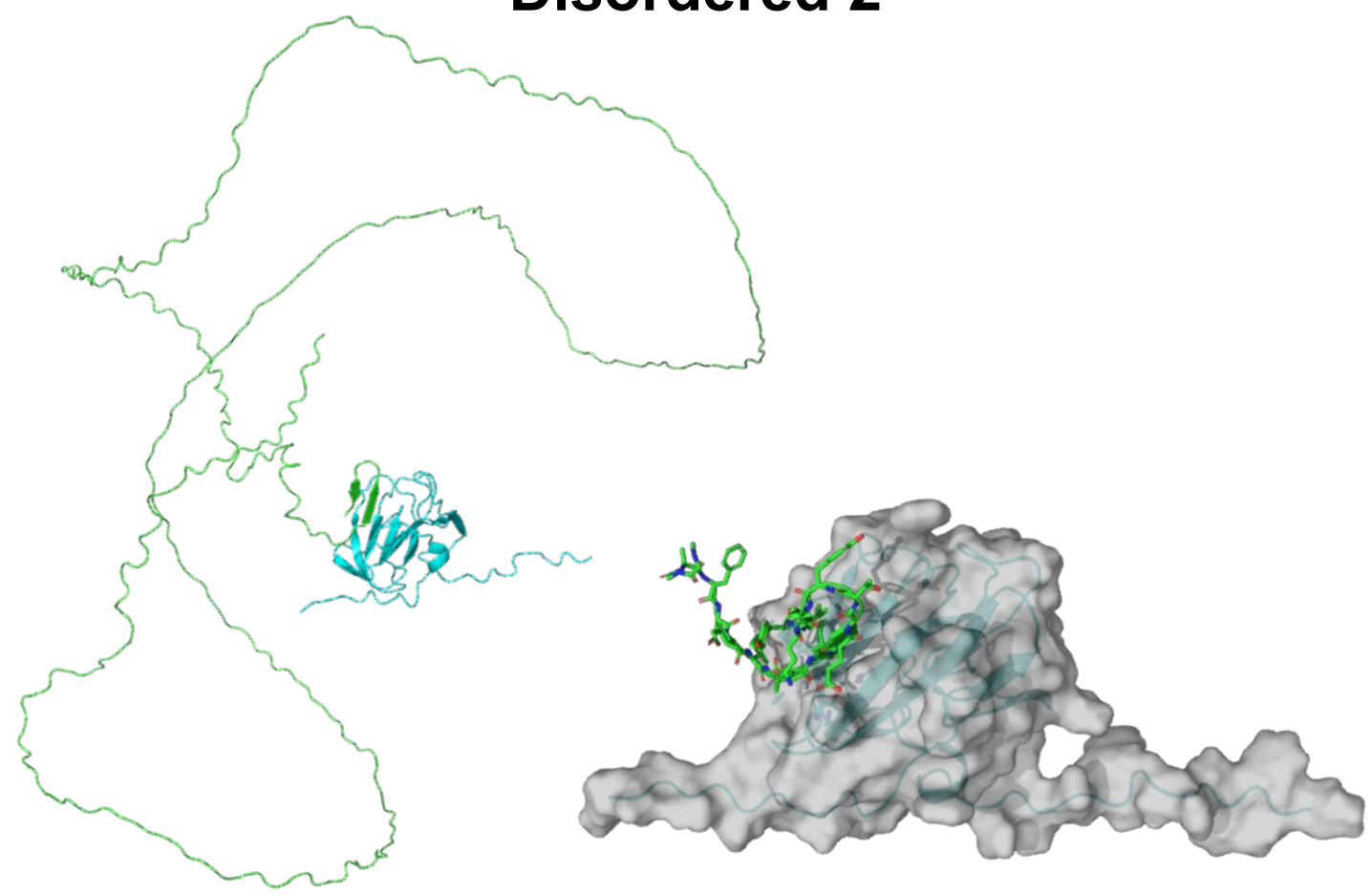

Disordered 3

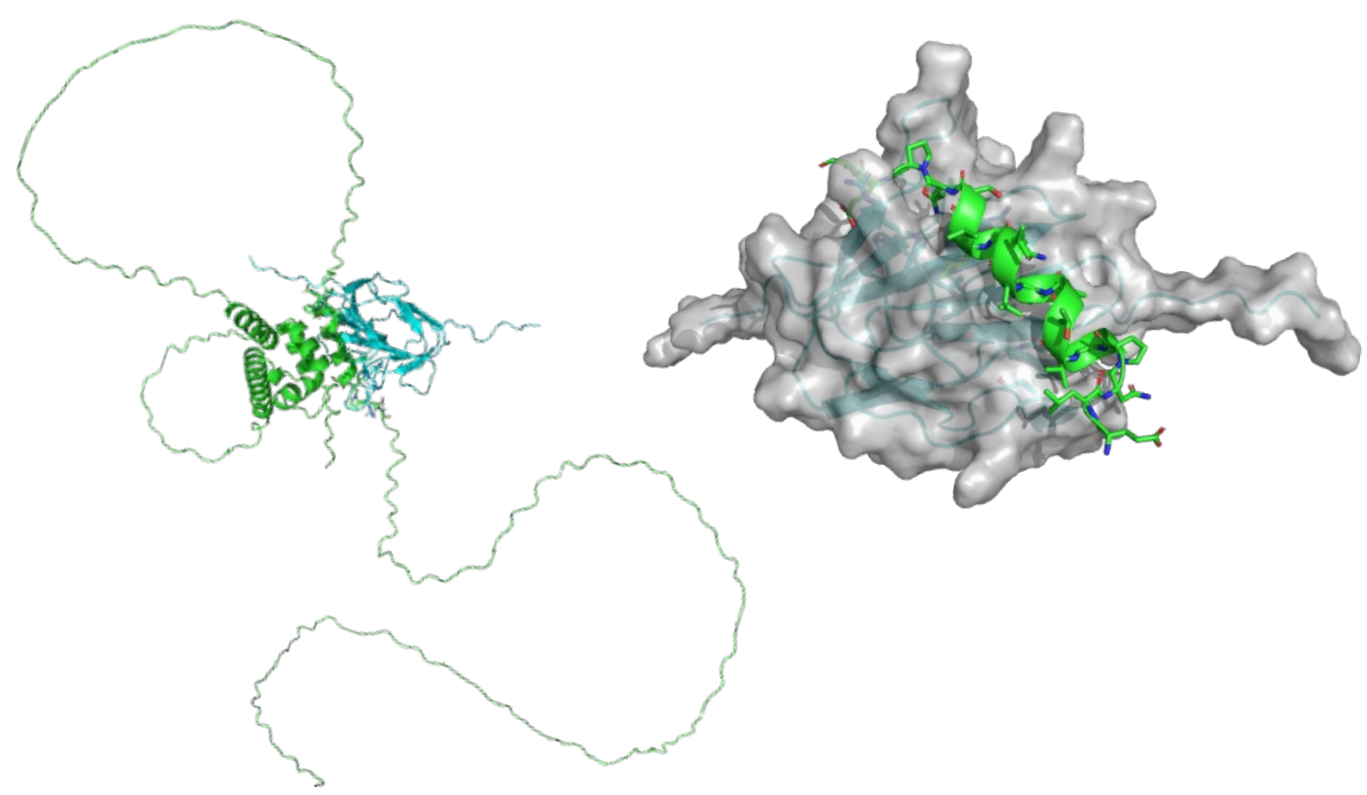

Supplementary Figure 4. Predicted interactions between CDK12 and LytM.
(**A**) AlphaFold generated scoring of predicted LytM interaction with different domains of CDK12. (**B**) Top scoring predicted interactions between each CDK12 domain and LytM are shown.

Supplementary Figure 5

A

Peptide	RK48		EV19		FA30		MK34		YV32	EL32
Binding partner	LytM	NlpD	LytM	NlpD	LytM	NlpD	LytM	NlpD	NlpD	NlpD
k_a ($M^{-1}s^{-1}$)	8453	2949	14780	6343	3449	4347	4268	1189	1338	478.3
k_d (s^{-1})	0.001754	0.00134	0.0006403	0.00101	0.000805	0.000576	0.001107	0.001759	0.001403	0.001695
K_D (μM)	0.208	0.454	0.043	0.159	0.233	0.133	0.259	1.479	1.049	3.544

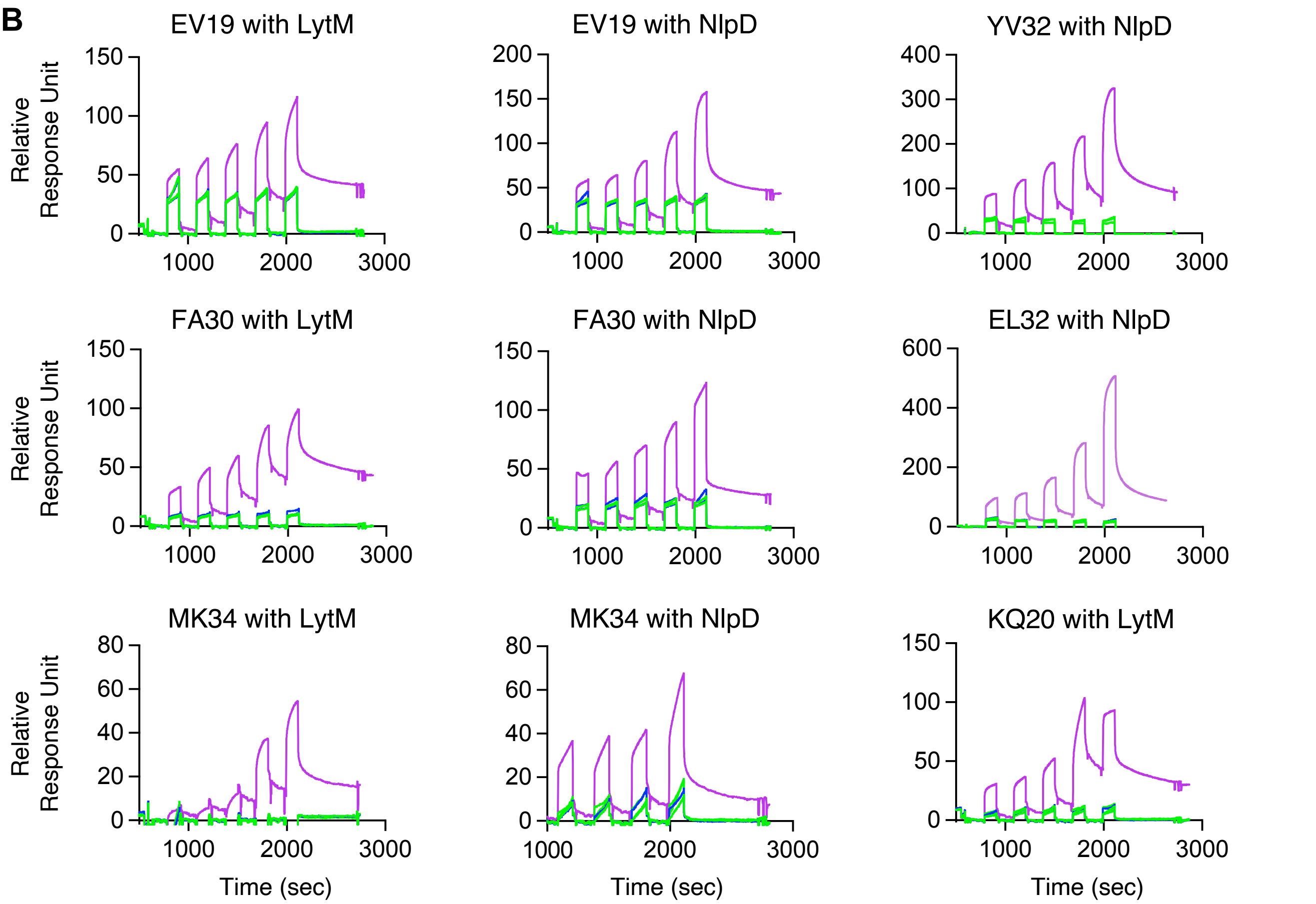

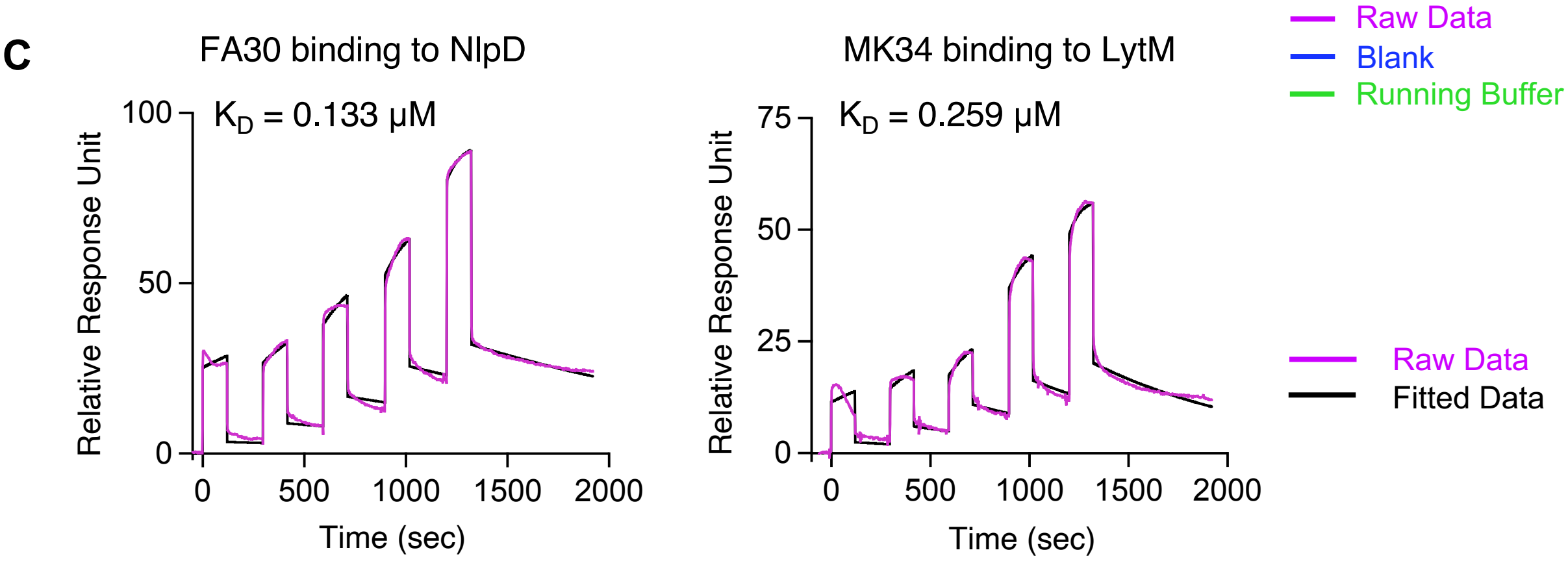

Supplementary Figure 5. Surface Plasmon Resonance binding analysis.
(**A**) Binding affinities calculated from rate constants obtained from data fitting to a 1:1 Langmuir binding model. (**B**) Raw SPR data obtained from the reference surface during each binding assay to the NTA sensor chip. This display captures the running buffer (green) and sample buffer responses (referred as blank, blue), commonly referred to as "bulk responses", to ensure reliable baseline corrections. Analyte-specific curves (magenta) highlight the functional differences among the proteins studied. (**C**) SPR binding analysis showing the dose-dependent interaction of NlpD and LytM with the FA30 and MK34 peptides, respectively. The His-tagged recombinant LytM and NlpD proteins were captured on Sensor Chip NTA and flooded with increasing concentrations of the peptides (magenta). Data was fitted to a 1:1 Langmuir binding model (black). Equilibrium dissociation constants (K_D) of bindings are indicated.

Supplementary Figure 6

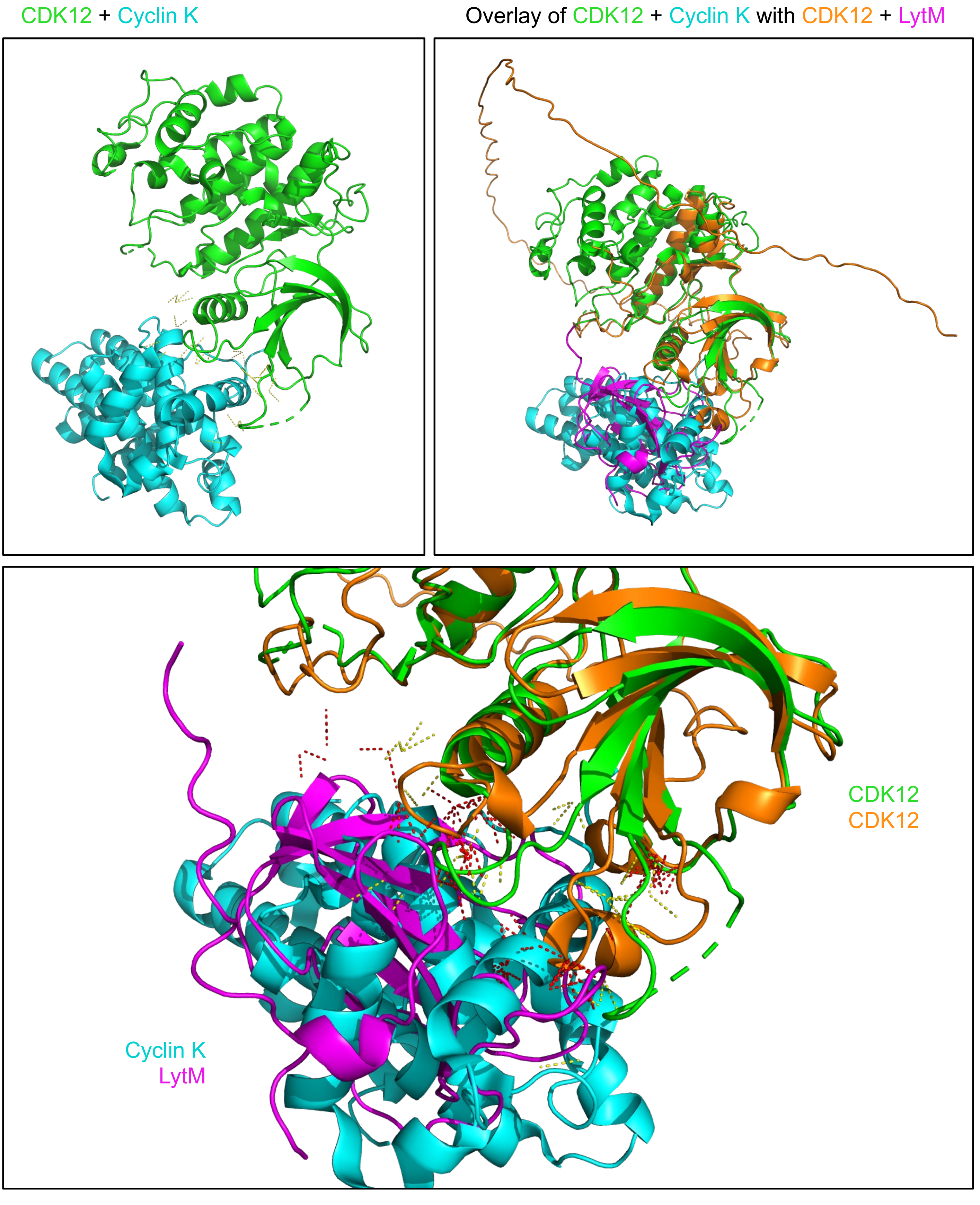

Supplementary Figure 6. Predicted CDK12 and LytM binding.
Overlay of the human CDK12:Cyclin K complex crystal structure (PDB 5ACB) with the modelled CDK12:LytM complex predicted by the AlphaFold multimer.

Supplementary Figure 7

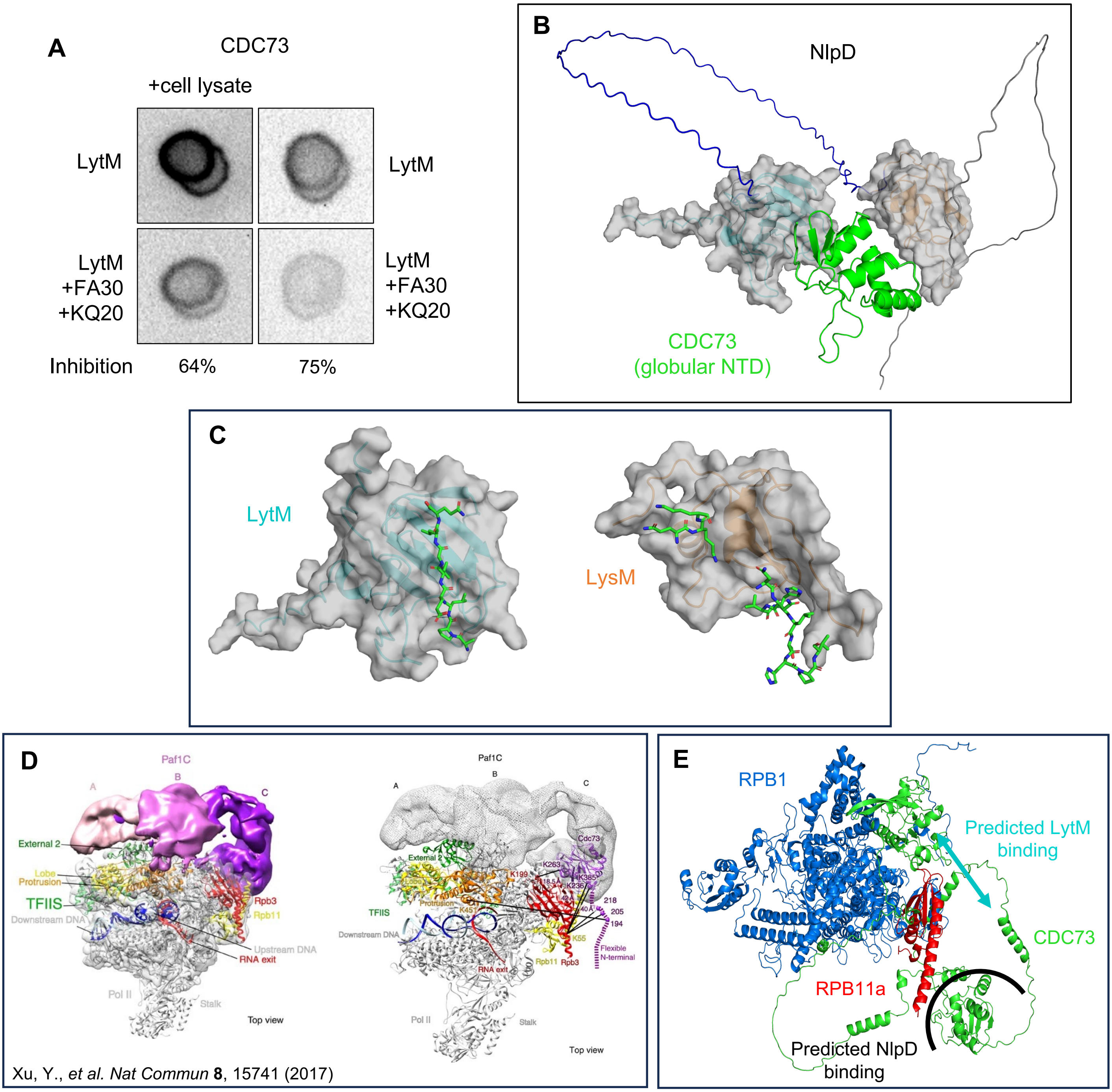

Supplementary Figure 7. Interactions between CDC73, LytM and NlpD.
(**A**) Dot blot analysis of LytM interaction with CDC73 from cellular extracts (A498 cells whole cell lysates). Binding of LytM to CDC73 (aa 298-327 and aa 392-411) is inhibited by the corresponding synthetic peptides (FA30 and KQ20). (**B**) AlphaFold generated structure of the globular N-terminal domain (NTD) of CDC73 (green) with interacting NlpD. (**C**) Detail of the predicted interaction between the LytM (cyan) and the LysM (orange) domains of NlpD with the globular N-terminal domain of CDC73. (**D**) Cryo-EM based assemblies of the Pol II complex, indicating the positions and interactions of PAF1C, RPB1 and RPB11a, found in the litterature. Figure from Xu *et al. Nat Commun*. **8**:15741 (2017), doi:10.1038/ncomms15741. (**E**) AlphaFold predicted CDC73, RPB1 and RPB11a complex assembly, indicating the predicted binding site of LytM (cyan) and NlpD (black). The predicted LytM binding to C-terminal globular domain and central disordered region of CDC73, could limit the flexibility of CDC73, not allowing it to effectively wrap around RPB1. The predicted NlpD binding to N-terminal globular domain of CDC73, could impede the interaction with RPB11a, interfering with anchoring of the PAF1C complex to the Pol II complex.

Supplementary Figure 8

A Scoring for LytM interaction with RPB1

Target region (residues)	pTM	ipTM	Rank (region)	Rank (protein)
**Helical / disordered 1-186**	0.438	0.101	4	26
	0.470	0.414	1	4
	0.437	0.108	3	24
	0.428	0.091	5	30
	0.435	0.195	2	17
**Globular 1 186-531**	0.372	0.115	3	23
	0.378	0.152	2	19
	0.389	0.285	1	12
	0.416	0.095	5	29
	0.373	0.096	4	28
**Globular 2 531-801**	0.555	0.489	2	2
	0.587	0.464	3	3
	0.605	0.592	1	1
	0.541	0.397	5	7
	0.529	0.403	4	5
**Globular / helical 801-1121**	0.469	0.104	2	25
	0.520	0.086	5	32
	0.495	0.123	1	21
	0.474	0.101	3	26
	0.505	0.087	4	31
**Globular 3 1121-1301**	0.473	0.168	5	18
	0.494	0.197	4	16
	0.540	0.288	1	11
	0.525	0.260	2	13
	0.519	0.244	3	14
**Globular 4 1301-1560**	0.379	0.137	2	20
	0.364	0.083	4	34
	0.367	0.080	5	35
	0.363	0.084	3	33
	0.376	0.199	1	15
**Repeats 1560-1970**	0.306	0.372	2	8
	0.295	0.399	1	6
	0.304	0.322	4	10
	0.293	0.359	3	9
	0.308	0.121	5	22

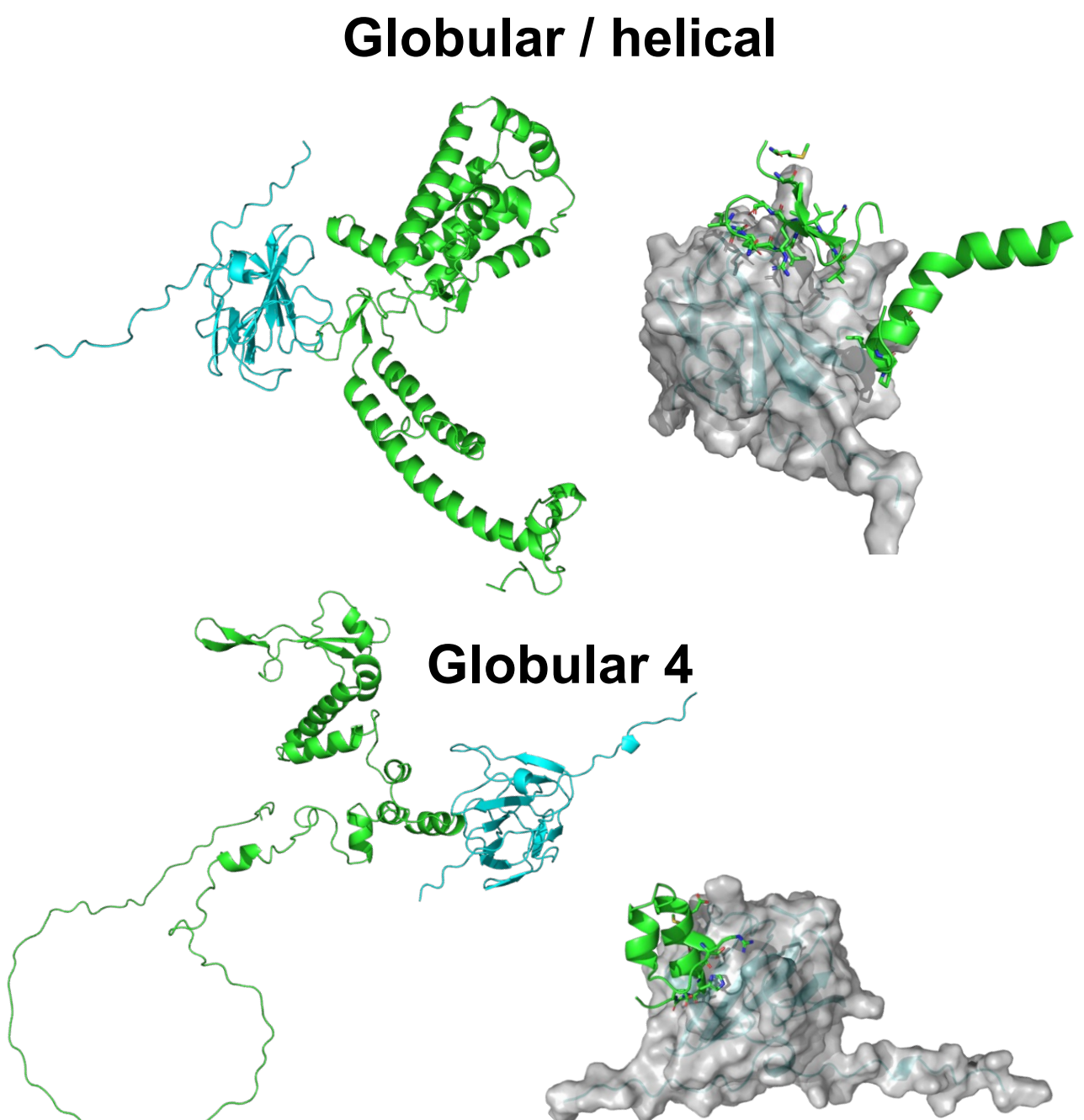

B Top ranked LytM interaction with RPB1

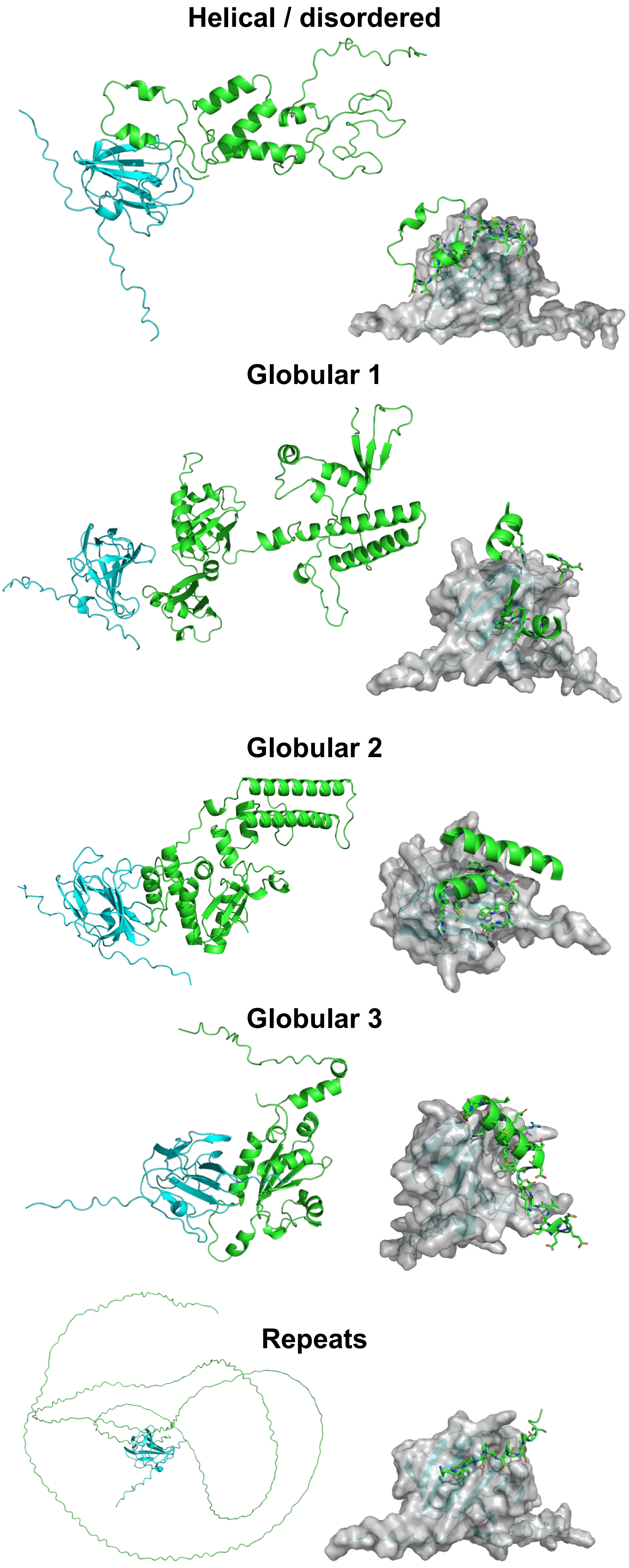

Supplementary Figure 8. Predicted interactions between RPB1 and LytM.
(**A**) AlphaFold generated scoring of predicted LytM interaction with different domains of RPB1. (**B**) Top scoring predicted interactions between each RPB1 domain and LytM are shown.

Supplementary Figure 9

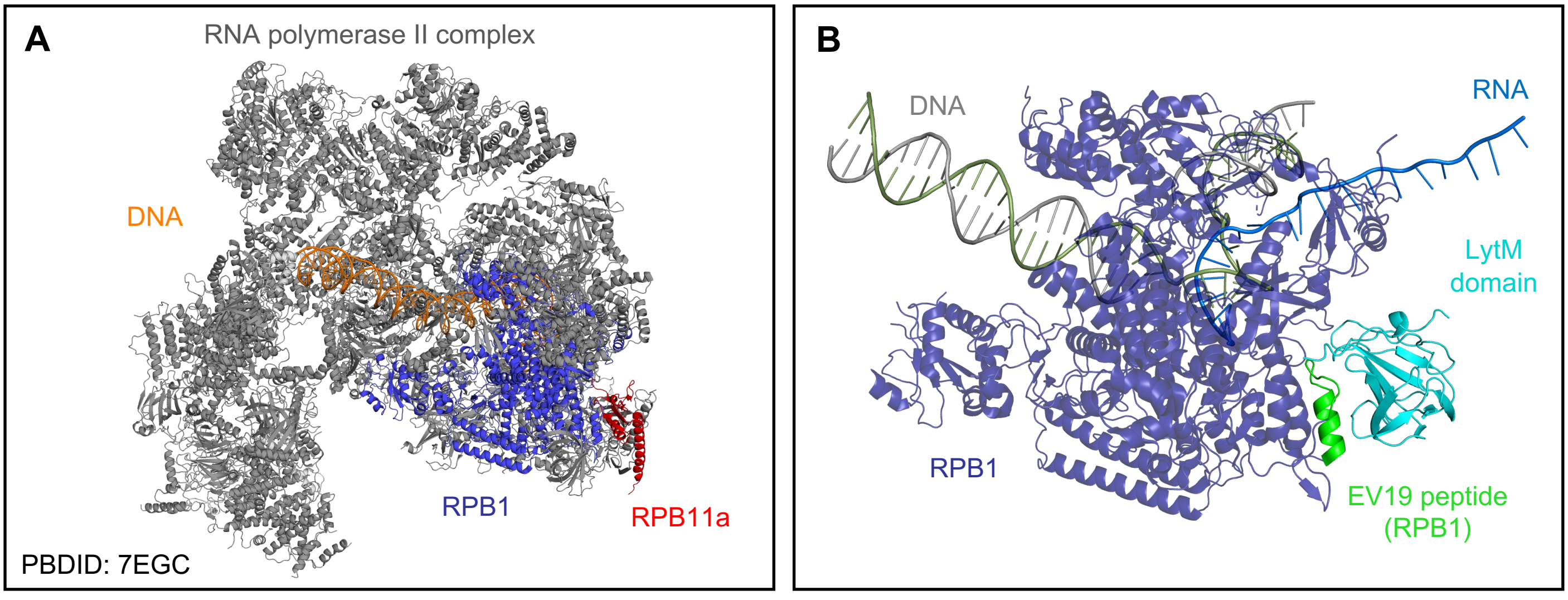

Supplementary Figure 9. Interactions between RPB1, LytM and RPB11a.
(**A**) Human RPB1:RPB11a complex assembly on DNA (PDB 7EGC). (**B**) Modelled RPB1:LytM complex predicted by AlphaFold multimer. For clarity, only the RPB1 region interacting with LytM (EV19) is shown in green.

Supplementary Figure 10

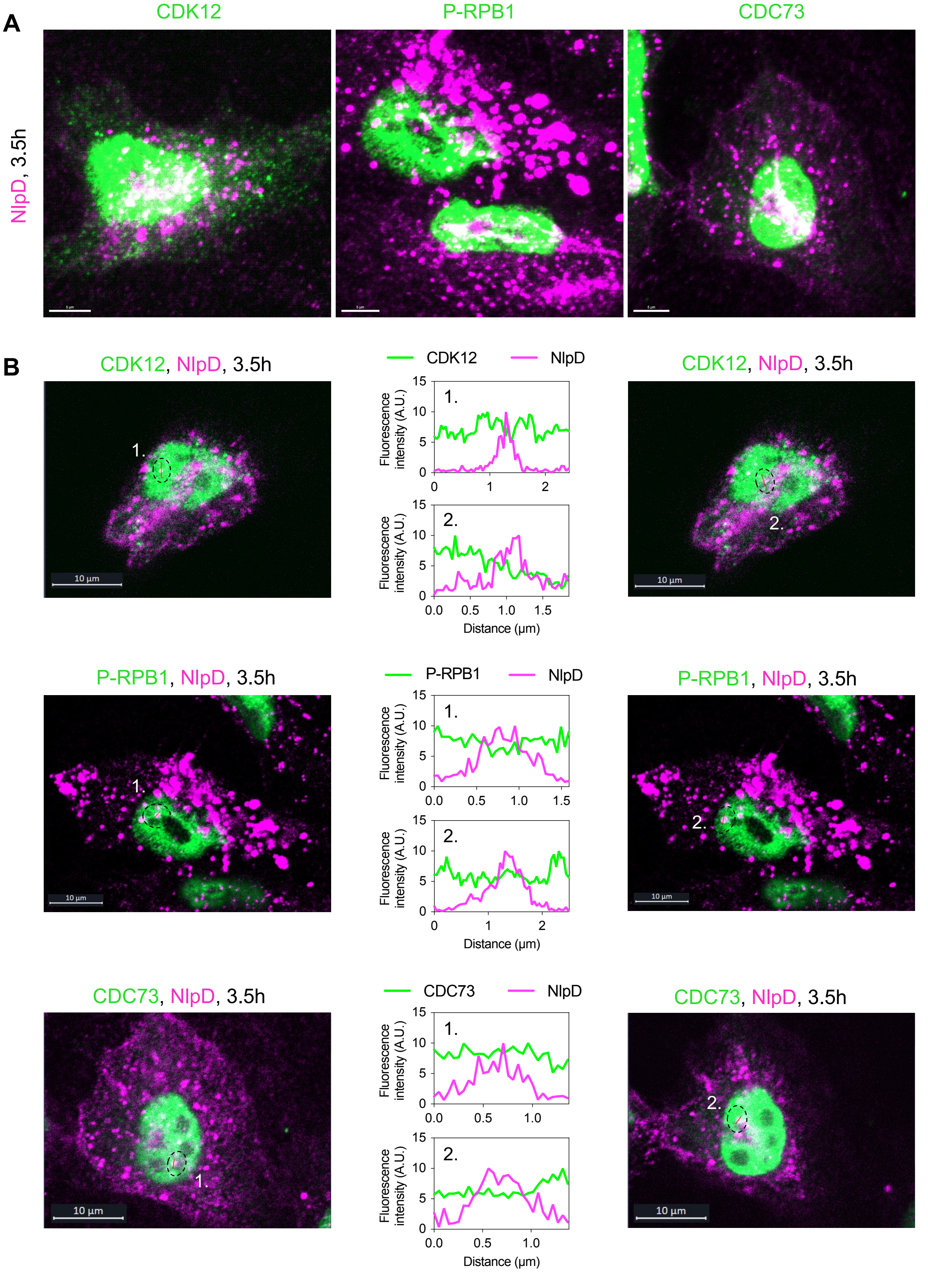

Supplementary Figure 10. Co-localization of rNlpD with cellular targets.
(**A**) Analysis of interactions between rNlpD (magenta) and cellular targets (green) CDK12, phosphorylated RPB1 and CDC73 by immunocytochemistry. (**B**) Co-localization of rNlpD with the cellular targets is verified by line scans. Quantification of two line-scans signal intensities for each cellular target. Separate line scans are indicated by the numbers. Scale bars 10 µm.

Supplementary Figure 11

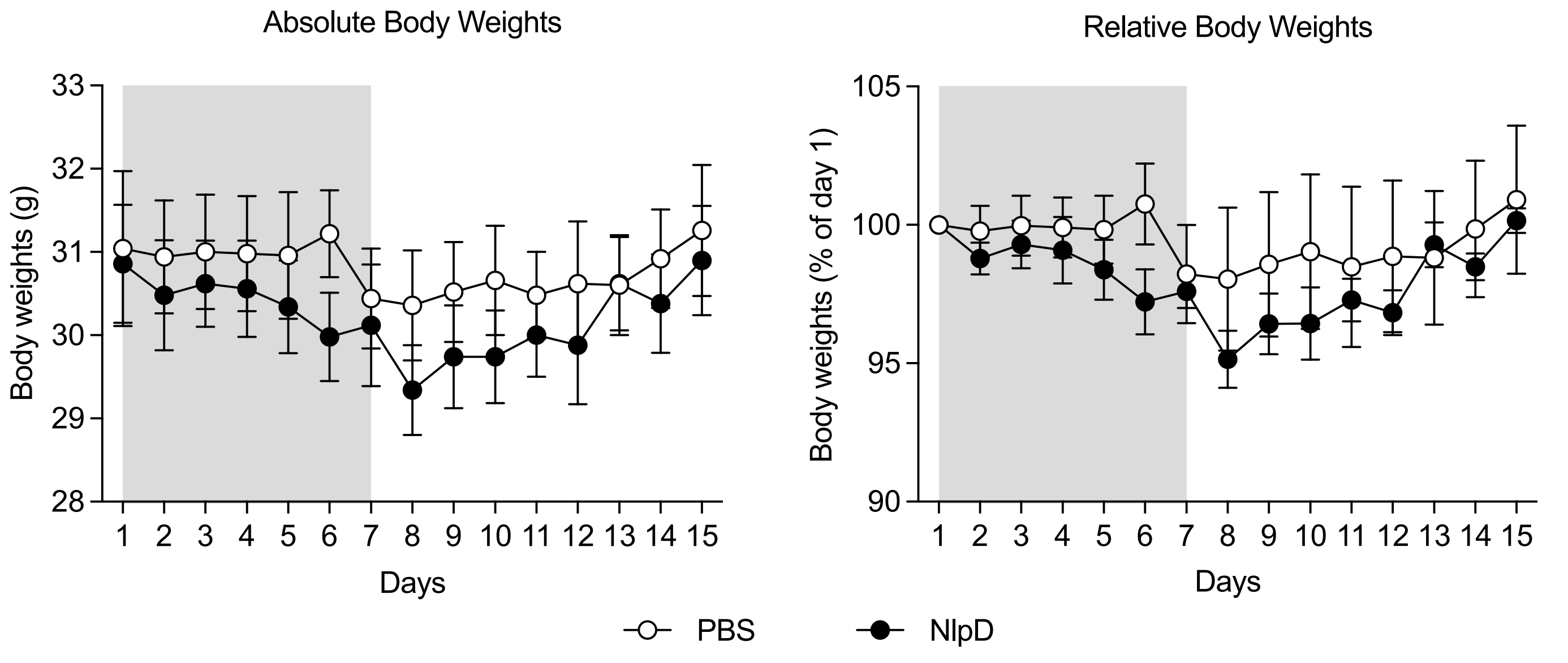

Supplementary Figure 11. Body weights following intravenous injection of NlpD. Seven weeks old female NMRI mouse were injected with NlpD (4.2 mg/kg, 0.84 mg/mL) or PBS (5 mL/kg). Injections were done intravenously, once daily for 7 days (grey in graphs). Animals were followed for 15 days. No overt clinical signs were apparent following administration of NlpD, expect for transient redness and swelling at the base of the tail, in three of five animals (between days 3 and 5). Swelling and redness dissipated when dosing ceased, expect for one animal on day 8. 2-way repeated measure ANOVA followed by Sidak multiple comparison tests.

Supplementary Figure 12

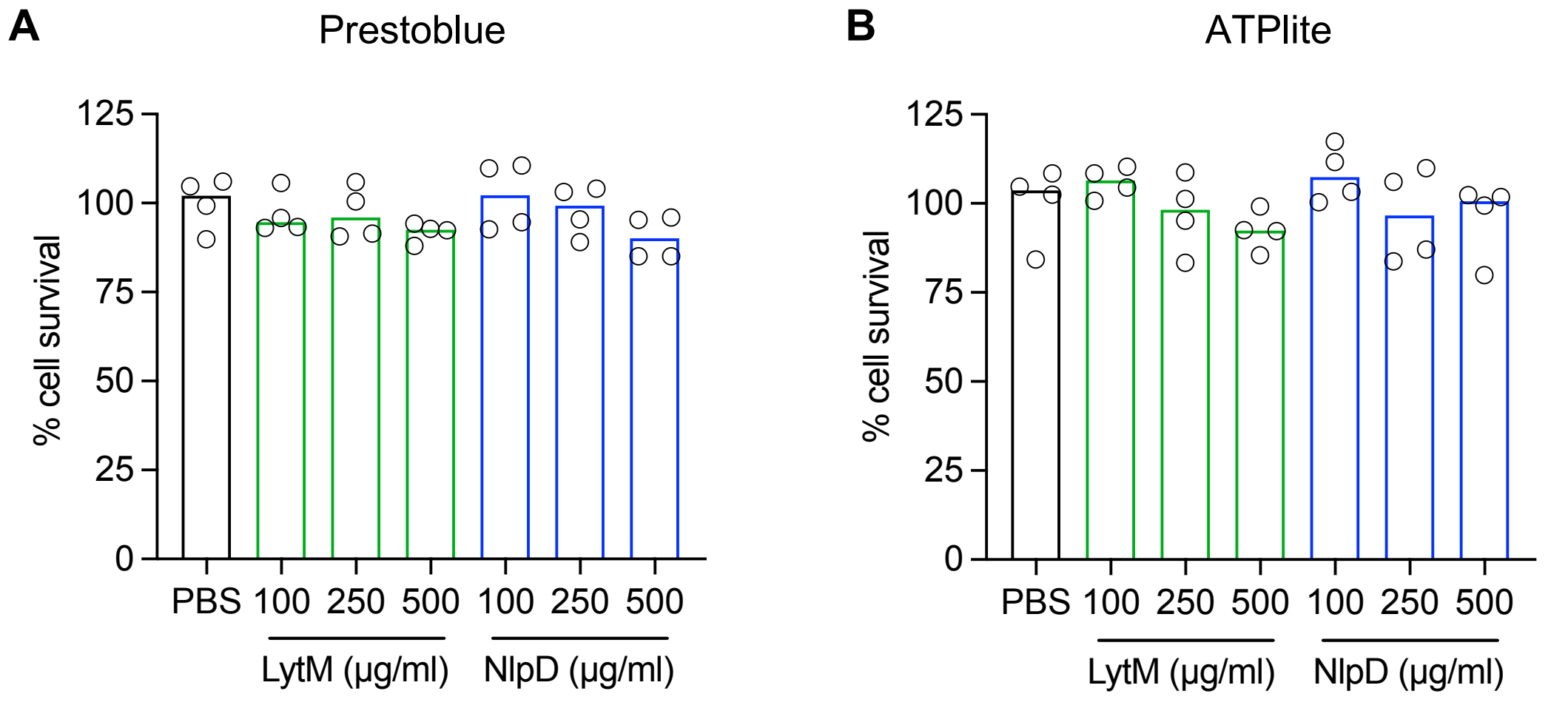

Supplementary Figure 12. Analysis of LytM and NlpD cytotoxicity.
Human lung carcinoma cells (A549) were treated with increasing concentrations of rNlpD and LytM. Cell viability was evaluated by Prestoblue assay (A) and ATP levels (ATPlite assay (B). No evidence of decreased cell viability was observed. Individual values and median, n = 2 replicates and n = 2 independent experiments. Kruskal-Wallis test with Dunn's multiple comparison compared to PBS control.

Supplementary Figure 13

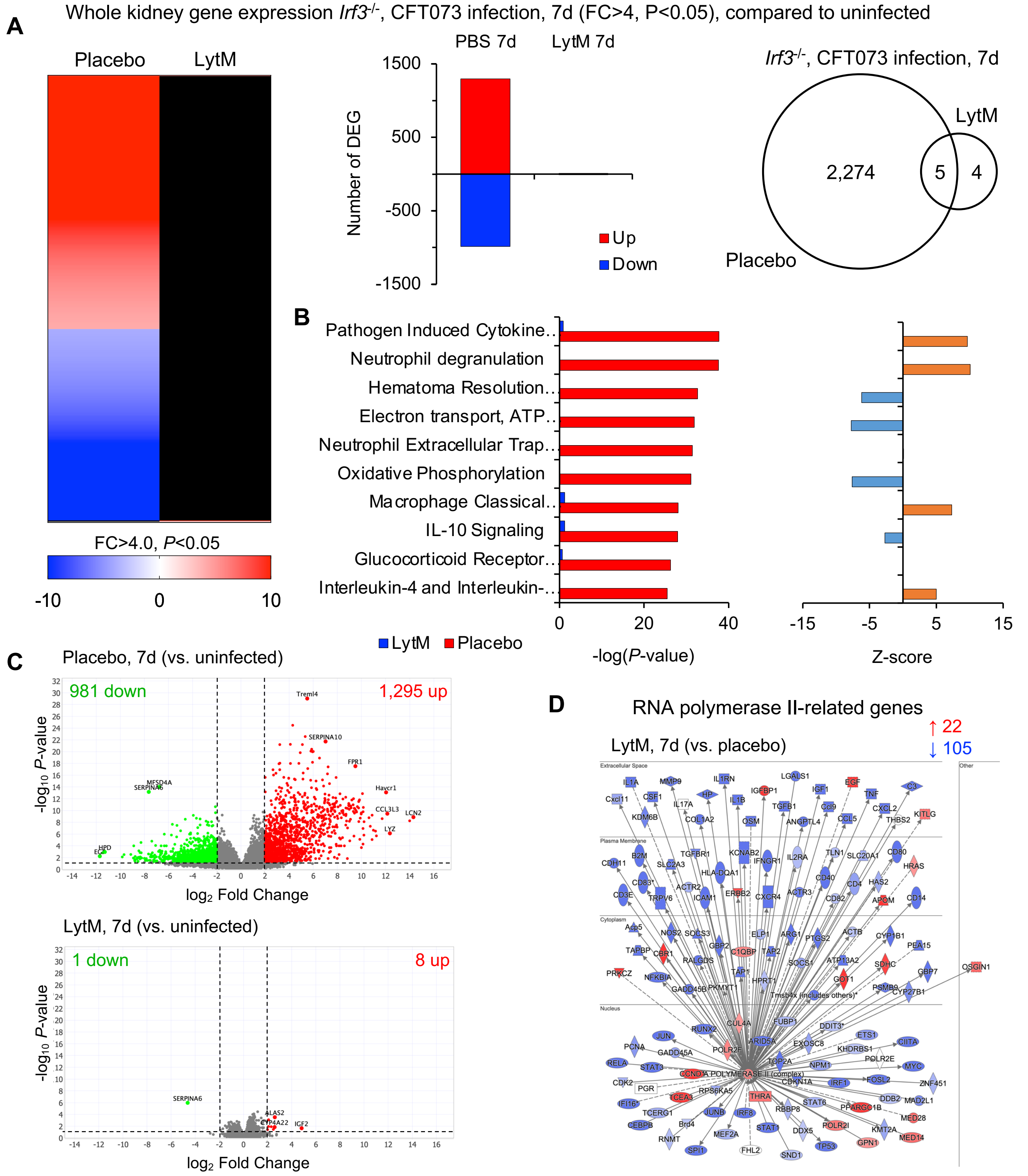

Supplementary Figure 13. Inhibition of the disease response defined by gene expression analysis. Total renal RNA isolated from infected mice or uninfected mice was subjected to genome-wide transcriptomic analysis. Disease-associated genes were identified by comparing infected mice to uninfected controls (heat map, cut off FC ≥ 4, P < 0.05, n = 2 per group; red, upregulated; blue, downregulated). (**A**) Inhibition by LytM treatment of the response to infection, shown as a marked reduction in the number of differentially expressed genes in treated mice compared to the placebo group. (**B**) Functional analysis identifying a strong activation of an innate immune response in the placebo group and a lack of response in the LytM-treated group. Activation Z-score are shown. (**C**) Volcano plots of all the detected genes (n = 19,863 genes) identifying genes differentially expressed in the treated and placebo groups (grey, no change; red, activated; green, inhibited). (**D**) Network analysis of the RNA polymerase II-related genes regulated by LytM treatment in the infected mice, genes were inhibited compared to the placebo group (n = 2 per group, seven days post infection compared to placebo; white, no change; red, activated; blue, inhibited).

Supplementary Figure 14

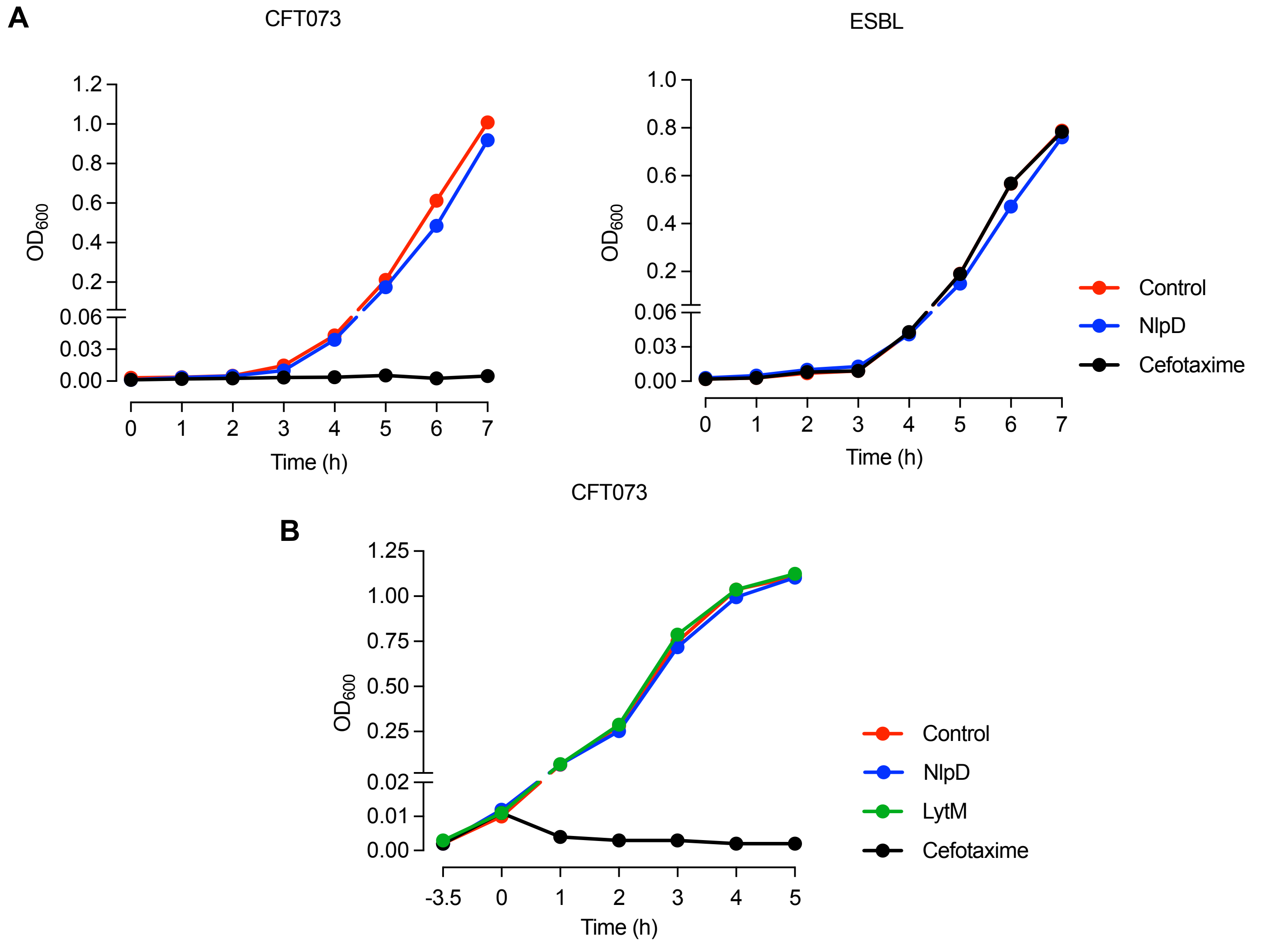

Supplementary Figure 14. Effect of NlpD and LytM on the bacterial growth in vitro.
(**A**) Lack of effect of NlpD treatment (106 µg) on growth of *E. coli* CFT073 and ESBL strains (10^4 cfu, 1 mL) cultured in LB media (37°C). (**B**) NlpD and LytM treatments did not kill *E. coli* CFT073 (10^4 cfu). Bacteria was allowed to grow for 3.5h in LB media before treatment with NlpD (106 µg) or LytM (43 µg) at time 0. Bacterial growth was monitored by measuring OD_{600} values every hour. Cefotaxime (250 µg) and PBS were used as controls. No difference in bacterial growth was observed compared to controls, while cefotaxime treatment inhibited the growth of CFT073 but not the ESBL strain.

Table S1. Predicted interaction scorings.

Target		Residues	LytM (ipTM)	LysM (ipTM)	NlpD (ipTM)
**CDK12**	NTD	1-726	0.361	0.156	
	Kinase domain	727-1019	0.598	0.232	0.408
	CTD	1020-1490	0.354	0.252	
**RPB1**	Region 1	1-359	0.233		
	Region 2	360-776	0.595	0.126	
	Region 3	777-1129	0.214		
	Region 4	1130-1463	0.275		
	Region 5	1464-1970	0.410		
**CDC73**	Full length		0.603	0.219	0.277
	NTD	1-118	0.444	0.301	0.271
	Central DR	119-356	0.306	0.106	0.204
	CTD	357-531	0.170	0.232	0.263
**RPB11a**	Full length		0.473		0.458
**LEO1**	Full length		0.389	0.190	
**PAF1**	Full length		0.405	0.337	
**CTR9**	Globular domain 1	1-487	0.209	0.536	
	Globular domain 2	488-811	0.285	0.213	
	CTD	812-1173	0.332	0.116	
**RTF1**	Full length		0.247	0.253	

Table S2. Toxicology results

	PBS (n = 5)		NlpD (n = 5)		P-value
	Mean	SEM	Mean	SEM	
**Hematology**					
WBC (10^9/L)	9.06	1.46	7.3	1.06	0.358
LYM (10^9/L)	7.86	1.32	6.22	0.93	0.340
LYM (%)	85.38	0.88	83.94	1.32	0.391
MON (10^9/L)	0.34	0.04	0.32	0.07	0.810
MON (%)	4.38	0.27	4.84	0.09	0.145
GRA (10^9/L)	0.86	0.11	0.76	0.12	0.556
GRA (%)	10.24	0.62	11.22	1.4	0.540
HGB (g/dL)	13.72	0.28	12.98	0.27	0.094
MCH (pg)	16.68	0.21	16.38	0.23	0.364
MCHC (g/dL)	34.74	1.07	35.74	0.35	0.400
RBC (10^{12}/L)	8.23	0.2	7.91	0.14	0.226
MCV (fL)	48.28	1.79	45.9	0.71	0.252
HCT (%)	39.64	0.88	36.34	0.92	0.032
RDW (fL)	31.56	0.98	30.22	0.53	0.263
RDW (%)	15.70	0.32	16.38	0.15	0.091
PLT (10^9/L)	942.6	43.66	895.2	69.14	0.578
MPV (fL)	4.64	0.1	4.70	0.17	0.769
**Clinical chemistry**					
ALB (g/L)	39.0	8.73	39.6	0.60	0.940
ALP (U/L)	94.8	2.67	107.8	7.07	0.124
ALT (U/L)	46.6	5.80	50.6	14.08	0.799
AMY (U/L)	857.2	35.01	840.6	26.88	0.717
TBIL (µmol/L)	6.4	0.24	6.4	0.24	>0.999
BUN (mmol/L)	7.96	0.91	6.06	0.51	0.106
CA (mmol/L)	2.58	0.02	2.71	0.04	0.020
PHOS (mmol/L)	2.56	0.07	2.63	0.05	0.439
CRE (µmol/L)	25.2	1.39	24.8	2.71	0.899
GLU (mmol/L)	12.46	0.97	10.52	0.39	0.101
Na^+ (mmol/L)	146.6	1.03	148.2	0.58	0.213
K^+ (mmol/L)	5.26	0.20	5.58	0.16	0.247
TP (g/L)	50.2	0.58	51.2	0.80	0.341
GLOB (g/L)	10.5	2.39	11.8	0.37	0.563
**Organ weights**					
Bladder (g)	0.02	0.00	0.02	0.00	>0.999
Spleen (g)	0.11	0.01	0.14	0.01	0.067
Kidneys (g)	0.33	0.02	0.33	0.02	>0.999
Liver (g)	1.50	0.05	1.57	0.06	0.396
Lungs (g)	0.25	0.01	0.25	0.02	>0.999
Heart (g)	0.14	0.01	0.16	0.01	0.195
Brain (g)	0.47	0.01	0.47	0.01	>0.999

WBC (white blood cell count), LYM (lymphocytes), MON (monocytes), GRA (granulocytes), HGB (haemoglobin), MCH (mean corpuscular haemoglobin), MCHC (mean corpuscular haemoglobin concentration), RBC (red blood cell count), MCV (mean corpuscular volume), HCT (hematocrit), RDW (red cell distribution width), PLT (platelet count), MPV (mean platelet volume), ALB (albumin), ALP (alkaline phosphatase), ALT (alanine transaminase), AMY (amylase), TBIL (total bilirubin), BUN (urea), CA (calcium), PHOS (phosphate), CRE (creatinine), GLU (glucose), Na^+ (sodium), K^+ (potassium), TP (total protein), GLOB (globulin)

Table S3. Top regulated genes in the placebo group.

Symbol	Entrez Gene Name	Placebo	NlpD	LytM	Location	Type(s)
Lcn2	lipocalin 2	1,833.0			Extracellular Space	transporter
Il1rn	interleukin 1 receptor antagonist	1,228.4			Extracellular Space	cytokine
S100a8	S100 calcium binding protein A8	1,208.5			Cytoplasm	other
Acod1	aconitate decarboxylase 1	1,166.5			Cytoplasm	enzyme
Csf3r	colony stimulating factor 3 receptor	1,128.5			Plasma Membrane	transmembrane receptor
Slc15a3	solute carrier family 15 member 3	1,125.5	6.8	6.5	Cytoplasm	transporter
Ccl3	C-C motif chemokine ligand 3	989.5			Extracellular Space	cytokine
Il36g	interleukin 36 gamma	827.2			Extracellular Space	cytokine
Saa3	serum amyloid A 3	804.3		-12.7	Extracellular Space	other
Clec4e	C-type lectin domain family 4 member E	756.3			Plasma Membrane	other
Slc22a7	solute carrier family 22 member 7	-184.9			Plasma Membrane	transporter
Slc22a22	solute carrier family 22 (organic cation transporter), member 22	-196.1			Plasma Membrane	transmembrane receptor
Cyp2d9	cytochrome P450, family 2, subfamily d, polypeptide 9	-204.3			Cytoplasm	enzyme
Ces1e	carboxylesterase 1E	-208.5			Cytoplasm	enzyme
C2orf80	chromosome 2 open reading frame 80	-269.3			Other	other
Ucp1	uncoupling protein 1	-291.9	-54.6	-566.3	Cytoplasm	transporter
Pvalb	parvalbumin	-673.2			Cytoplasm	other
Hpd	4-hydroxyphenylpyruvate dioxygenase	-675.8			Cytoplasm	enzyme
Inmt	indolethylamine N-methyltransferase	-783.4			Cytoplasm	enzyme
Dnase1	deoxyribonuclease 1	-984.6			Extracellular Space	enzyme

Table S4. Predicted LytM binding peptides.

Target		Peptide	Sequence	Residues
**CDK12**	Kinase domain	RK48	RLDNEKEGFPITAIREIKILRQLIHRSVVNMKEIVTDKQDALDFKKDK	759-806
	Kinase domain	VI20*[1]	VRLDNEKEGFPITAIREIKI	758-777
	Kinase domain	IL20*[1]	IVTDKQDALDFKKDKGAFYL	792-811
	NTD (DR)	ID20	IKPLVEYDDISSDSDTFSDD	67-86
	NTD (DR)	WY24	WGKRCVDKFDIIGIIGEGTYGQVY	719-742
	CTD (DR)	EP21	ELNPAVTAALLQLLSQPEAEP	1292-1312
**RPB1**		EV19	ERGEVMNLLMFLSTWDGKV	556-574
		EG29	EGGEEMDNKFGVEQPEGDEDLTKEKGHGG	155-183
**CDC73**	Central DR	FA30*[2]	FKGKEETEGFKIDTMGTYHGMTLKSVTEGA	298-327
	CTD	KQ20*[2]	KKKQGCQRENETLIQRRKDQ	392-411
	NTD/central DR	EE30	EASTSASIDRSAPLEIGLQRSTQVKRAADE	101-130
	NTD	RT13	RSAPLEIGLQRST	110-122
	Central DR	LR22	LKQRSFVDAEVDVTRDIVSRER	208-229
	CTD	QI9	QMQPGGTAI	411-419

*Both are predicted to be required for binding

Table S5. Predicted NlpD binding peptides.

Target		Peptide	Sequence	Residues
**CDC73**	NTD	MK34	MADVLSVLRQYNIQKKEIVVKGDEVIFGEFSWPK	1-34
**CDC73**	NTD	YV32	YYTLDSILFLLNNVHLSHPVYVRRAATENIPV	54-85
**CDC73**	NTD	EL32	EASTSASIDRSAPLEIGLQRSTQVKRAADEVL	101-132

SUPPLEMENTARY METHODS

In silico modelling

Each target protein sequence (UniProt ID: Q9NYV4, P24928, Q6P1J9 and P0ADA3 for CDK12, RPB1, CDC73 and NlpD, respectively) was divided into segments based on tertiary structure previously determined either experimentally or by AlphaFold and then submitted to CoLabFold using AlphaFold2 multimer protocol (1) together with the LytM or NlpD sequence. The 5 top scoring complexes for each search were then visually evaluated using PyMOL (the PyMOL Molecular Graphics System, Version 2.5, Schrodinger, LLC.) filtering out complexes formed by either the LytM purification tag or those termini arising from dividing the sequence. After this the complex with the best ipTM value for each sequence segment was used as template for the synthetic peptides used in the subsequent binding assays.

Recombinant proteins

The recombinant construct used in this study (rNlpD) is based on the NlpD sequence from *E. coli* 83972 and has a C-terminal polyhistidine-tag (His-tag). Recombinant 6xHis-tagged NlpD (rNlpD), LytM, LysM, LytM with disordered region (LytM+DR) and LysM with disordered region (LysM+DR) were expressed in *E. coli* using the pET30a expression vector and TB fermentation (custom ordered from GenScript). rNlpD was obtained from inclusion bodies using 2-step purification by Ni column and Superdex 200 column. LytM and LysM were obtained from supernatant of cell lysate by Ni column. LytM+DR and LysM+DR were obtained from supernatant of cell lysate using 2-step purification by Ni column and Chromdex 200 column or Superdex 75, respectively.

All proteins were all above 90% purity as estimated by analysis of Coomassie-stained SDS-PAGE gels under reducing conditions (certificated of analysis provided by supplier, GenScript). Purity and molecular size of all recombinant proteins were verified by Western blot using mouse anti-His monoclonal antibody (GenScript Cat# A00186, RRID:AB_914704). In house, recombinant proteins were run under reducing conditions on 4-12% Bis-Tris SDS-PAGE gel followed by Coomassie staining. Endotoxin levels were <10 EU/mg (rNlpD) and <0.3 EU/mg (LytM).

For live imaging of treated cells and in vivo imagining experiment, recombinant proteins were N-terminally labeled with AZ647 NHS Ester (VectorLabs Fluoroprobes Cat# 1121-2), as per the manufacturer's instructions. Free dye was removed using dialysis cassettes (D-Tube Dialyzer Midi, Merck-Millipore Cat#71506) followed by centrifugation with centrifugal filter units (Amicon Ultra-15, Merck-Millipore Cat# UFC901024). The concentration of the labeled proteins was determined using NanoDrop 2000c (Thermo Fisher Scientific).

Cell culture

Human kidney carcinoma cells (A498, ATCC Cat#HTB-44, RRID:CVCL_1056) and human lung carcinoma cells (A549, ATCC Cat#CCL-185, RRID:CVCL_0023) were cultured in RPMI-1640 supplemented with 1 mM non-essential amino acids, 1 mM sodium pyruvate, and 10% fetal bovine serum (FBS), at 37°C with 5% CO_2. Depending on the experiment requirement, cells were cultured overnight in 10 cm diameter dish (4×10^4 cells per dish) or 8-well ibidi chamber slides (1×10^5 cells per well) pre-treated with poly L-Lysine, in medium supplemented with 10% FBS. Prior to treatment with recombinant proteins, cells were washed and kept in serum-free complete media.

Dot blots

For whole cell lysates (WCL), untreated A498 cells were lysed using NP-40 lysis buffer (20 mM Tris HCl pH 8.0, 100 mM NaCl, 1 mM EDTA, 0.5% NP-40) supplemented with phosphatase and protease inhibitors (both 1x, Roche Diagnostics). Cells were then scraped into tubes and subjected to thermal shock induction by freezing on dry ice and thawing at room temperature, for three cycles. Following thermal shock, cell lysate was centrifuged at 16,100 g at 4°C for 20 minutes, and supernatant was retained. Protein concentrations were then measured by Nanodrop using the Pierce 660 nm protein assay reagent (ThermoFisher Scientific) according to the manufacturer's instructions, using bovine serum albumin (BSA) as a standard.

Recombinant proteins (322 picomoles) were blotted onto a 0.1 μm nitrocellulose membrane and air dried. The membrane was then blocked with 5% BSA for 1h. Subsequently, the membrane was incubated with 300 μg WCL, overnight. Washing

steps were performed three times with PBS 0.1% Tween 20 (PBS-T) for 10 minutes each on a shaker. Primary antibody incubation was conducted for 1h30 at room temperature using anti-CDK12 (CrkRS, 1:1,000, Abcam Cat# ab57311, RRID:AB_941171), anti-Pol II (RPB1, 1:500, Santa Cruz Biotechnology Cat# sc-899, RRID:AB_632359) or anti-CDC73 (HRPT2, 1:500, Biorbyt Cat# orb101354, RRID:AB_3665791) antibodies in 5% BSA/PBS-T. After washing with PBS-T, membranes were probed with horseradish peroxide-conjugated anti-mouse (1:4,000, Agilent Dako Cat# P0260, RRID:AB_2636929) or anti-rabbit (1:4,000, Bio-Rad Cat# 1706515, RRID:AB_11125142) antibodies in 5% BSA/PBS-T for 1h at room temperature. Following additional washing steps, chemiluminescent detection was carried out by incubating the membrane for 5 minutes with an ECL substrate (GE Healthcare), and signal capture was done using the ChemiDoc XRS+ imaging system (Bio-Rad).

For peptide inhibition experiments, LytM (322 picomoles) was pre-incubated with target peptides (1.61 nanomoles each) at room temperature for 2 hours. The LytM-peptides mix was then blotted on the membrane as previously described, followed by incubation with WCL and antibody detection.

Western blotting

Treatment groups received 6.5 µM recombinant NlpD, LytM, or LysM proteins for 3 hours, while control cells received vehicle buffer treatment. Cell fractionation of cellular compartments was performed using Qproteome Cell Compartment kit (Qiagen) according to the manufacturer´s instructions. WCL were collected as above, using NP-40 lysis buffer. Proteins were then resolved under reducing conditions on 4-12% Bis-Tris SDS-PAGE gel (ThermoFisher), blotted onto PVDF membranes (Bio-Rad), and stained with anti-NlpD (1:10,000 rabbit anti-sera custom-made, Innovagen, project# 11358)(2), anti-β-actin (1:4,000, Sigma-Aldrich Cat#A5441, RRID:AB_476744) or anti-histone H3 (1:4,000, abcam Cat#ab1791, RRID:AB_302613) antibodies in 5% non-fat dry milk (NFDM) overnight. Membranes were washed with PBS-T followed by incubation of HRP-conjugated anti-rabbit (1:4,000, Bio-Rad Cat# 1706515) or anti-mouse (1:4,000, Agilent Dako Cat# P0260) antibodies. Imaging was performed with

an ECL substrate (GE Healthcare) using the ChemiDoc XRS+ system (Bio-Rad). Protein bands were quantified by ImageJ.

Live cell imaging and surface reconstruction

For live cell uptake, cells grown in ibidi chamber slides ($1x10^5$ cells) pre-treated with poly L-Lysine overnight. Cells were exposed to N-terminal AZ647 (Click Chemistry Tools) labelled NlpD or its domains (LytM and LysM) (6.5 µM) for 1- and 3.5-hour interval in live cell imaging buffer (ThermoFisher Scientific). Nuclei were stained with Hoechst 33342 (ThermoFisher Scientific) for 10 min. Cells were immediately transferred to a confocal microscope (LSM900, Carl Zeiss) for imaging. Quantification of uptake was performed using Fiji software 1.46r (NIH).

3D rendering of the fluorescent images of cellular uptake were done using Imaris software 627 (BitPlane, v.9.9) from the whole volume z-stacks. The nuclear surface was made transparent to visualization the nuclear and peri-nuclear distribution of rNlpD and the domain peptides.

Immunocytochemistry

Treatment groups received 6.5 µM recombinant NlpD, LytM, or LysM proteins, or 5,6-Dichlorobenzimidazole 1-β-D-ribofuranoside (DRB, 40-60 µM, Merck-Millipore Cat# D1916) for 3 hours (3), while control cells received vehicle buffer treatment. Cells were treated and then fixed with 4% paraformaldehyde for 20 minutes at room temperature. After washing with PBS, cells were permeabilized for 3 min with 0.05% Triton X-100 and 0.3% NP-40 in PBS. Following another wash, cells were incubated for 1h at room temperature with blocking buffer (0.025% Triton X-100, 0.025% NP-40, and 4% goat serum in PBS). Cells were then incubated with anti-phospho RPB1 (1:500, Merck-Millipore Cat# 04-1571, RRID:AB_11212363) overnight. After washing, cells were incubated for 1 hour with Alexa Fluor 647 goat anti-rat antibodies (1:200, Thermo Fisher Scientific) and stained with Hoechst 33342 (1:2,000, Thermo Fisher Scientific), followed by three PBS washes and three distilled water washes. Finally, slides were mounted with ProLong Glass Antifade Mountant and CoverGrip Coverslip Sealant.

Imaging was done using a confocal microscope (LSM900, Carl Zeiss), with visualization performed using Zen Blue edition 3.1 software and Imaris Viewer 9.9.1.

Cell viability assays

The luminescence-based ATPlite kit (Perkin Elmer, Cat# 6016947) and the Prestoblue cell viability assay (Thermo Fisher Scientific, Cat# A13262) were used. A549 human lung epithelial cells (5 x 10^4 cells/well) were seeded in serum-free RPMI-1640 medium on 96-well plates, treated with NlpD or LytM at different concentrations (100, 250 and 500 µg/mL) and incubated for one hour. Afterward, FCS was added at a concentration of 5%, and the cells were continuously incubated for two hours at 37°C, at which time the two assays were carried out according to the manufacturers' instructions. Luminescence and fluorescence were measured using a microplate reader (Sense, Hidex). The experiments were performed in duplicates and repeated twice.

Synthetic peptides

Peptide synthesis was done by ProteoGenix. Mass spectrometry (MS) and high-performance liquid chromatrography (HPLC) quality control were performed. Lyophilized peptides were reconstituted in PBS to a final concentration of 2 mg/mL. A list of peptides used in this study is given in tables S4 and S5.

Surface plasmon resonance analysis

Real time biomolecular interaction analysis was performed by surface plasmon resonance (SPR) technology using a Biacore X100 instrument (Cytiva Life Sciences) loaded with a Sensor Chip NTA (Cytiva Life Sciences Cat# BR100034). All experiments were carried out at 25 °C with a constant flow rate of 30 µL/minute with 0.1 M HEPES, 1.5 M NaCl, 0.03 M EDTA and 0.5% v/v Surfactant P20 (Cytiva Life Sciences Cat# BR100826). A cell without nickel loading served as reference for nonspecific binding of proteins and chemicals. Biacore X100 control software was programmed to first prime the instrument with running buffer (0.1 M HEPES, 1.5 M NaCl, 0.03 M EDTA and 0.5% v/v Surfactant P20). $NiCl_2$ was flowed to saturate the

NTA with nickel ions, followed by a 3 mM EDTA wash. His-tagged NlpD (0.5 µM) and LytM (0.2 to 0.4 µM) ligands were prepared in running buffer and injected over the nickel activated sensor surface for capture. Peptide sample was injected over the captured His-tagged ligand with five different dilutions. Finally, the chip was regenerated with injection of 350 mM EDTA. Binding affinities were calculated from rate constants obtained from fitting blank subtracted data to a 1:1 Langmuir binding model.

In vivo fluorescence imaging of LytM

C57BL/6 mice were treated by intraperitoneal injections of N-terminally AZ647 labelled LytM (42.6 µg, 0.36 mg/mL), control mice received the same volume of PBS (118 µL). Organ and blood samples were collected after 24 hours or 48 hours and imaged using an IVIS Spectrum imaging system (Perkin Elmer). The LytM fluorescence signal was acquired at an excitation wavelength of 680 nm and fluorescence intensity was quantified using regions of interest in the IVIS software.

Bacterial growth analysis

E. coli CFT073 and the ESBL-producing *E. coli* strain (10^4 cfu) were cultured in 200µL LB media in the presence of NlpD (106 µg), cefotaxime (250 µg) or buffer control, and incubated at 37°C for 1 hour then transfer to 800µL LB media and incubated at for 7 hours. Alternatively, *E. coli* CFT073 (10^4 cfu) was cultured LB media for 3.5 hours prior to addition of NlpD (106 µg), LytM (43 µg), cefotaxime (250 µg) or buffer. OD_{600} were measured every hour to follow bacterial growth.

Murine urinary tract infection models

Female C57BL/6J *Asc*$^{-/-}$ or C57BL/6J *Irf3*$^{-/-}$ mice, aged 9–15 weeks, were used in this study. The *Asc*$^{-/-}$ mice were initially supplied by J. Tschopp's laboratory at the Department of Biochemistry, University of Lausanne, and the Institute for Arthritis Research (aIAR). The *Irf3*$^{-/-}$ mice were generously provided by the Riken Bioresource Center in Japan, with permission from T. Taniguchi (4). All mice were bred and housed

in a specific pathogen-free animal facility (Lund University, Lund, Sweden), in individual ventilated cages (IVCs), with unrestricted access to food and water.

Mice were intravesically infected with *E. coli* strain CY17 (5), CFT073 (6) or an ESBL-producing clinical isolate (7) (1×10^8 CFU in 50 µL PBS) through a soft polyethylene catheter (outer diameter 0.61 mm, Clay Adams), after intraperitoneal injection of a cocktail of ketamine (1.48 mg in 100 µL of 0.9% NaCl solution, Intervet) and xylazine (0.22 mg in 100 µL of 0.9% NaCl solution, Vetmedic). Mice were treated with rNlpD protein or LytM (100 µL, 27.13 µM in PBS) by intraperitoneal injection, 30 minutes after intravesical bacterial infection and daily, for up to 7 days. Placebo control mice received the same volume of buffer (50 mM Tris-HCl, 150 mM NaCl, 10% Glycerol) in PBS.

Urine samples were obtained at regular times after infection (24 hours, three days, five days and seven days). Viable counts were determined by growth on TSA plates (37°C, overnight) after serial dilutions as appropriate. Neutrophils in uncentrifuged urine were counted using a hemacytometer chamber. For outcome assessment and data analysis, investigators were blinded to what treatment animals received.

Animals were sacrificed using an overdose of isoflurane anesthesia (Dechra, Cat# 200-129), 24 hours or 7 days after infection. Bladders and kidneys were aseptically removed; macroscopic pathology was documented by photography.

Toxicology

For GLP study, performed by Scantox (formerly Adlego), Sweden, seven weeks old female NMRI mouse were injected with NlpD (4.2 mg/kg, 0.84 mg/mL) or PBS (5 mL/kg). Injections were done intravenously, once daily for 7 days and animals were followed for 15 days. Animal weights and health status were recorded daily, and organs were collected and weighed at Day 15.

Whole-genome transcriptome analysis

Total RNA was extracted from murine kidneys with the RNeasy Mini Kit (Qiagen Cat# 74104) after disruption in RLT buffer supplemented with β-mercaptoethanol (1%)

using CK28 Precellys Lysing kits (Bertin Technologies) and Tissuelyser (Qiagen). 10 ng of total RNA was amplified and fragmented using the GeneChip 3' IVT Pico labeling kit, followed by hybridization to Clariom S Mouse HT arrays (all ThermoFisher Scientific). Sample were processed in the GeneTitan System as per manufacturer's instruction (ThermoFisher Scientific). Data were normalized using Robust Multi Average implemented in Transcriptome Analysis Console software (v.4.0.1.36, Applied Biosystems, ThermoFisher Scientific). Relative expression was analyzed by ANOVA using the empirical Bayes method, and genes with an absolute fold change ≥ 6 or 4 and $P < 0.05$ were considered differentially expressed. Differentially expressed genes were functionally analyzed using Ingenuity Pathway Analysis software (IPA, Qiagen) and heatmaps were constructed in GraphPad Prism version 10.

REFERENCES

1. Mirdita, M, Schutze, K, Moriwaki, Y, Heo, L, Ovchinnikov, S, Steinegger, M. ColabFold: making protein folding accessible to all. Nat Methods. 2022;19(6):679-82; DOI:10.1038/s41592-022-01488-1.
2. Ambite, I, Filenko, NA, Zaldastanishvili, E, Butler, DS, Tran, TH, Chaudhuri, A, Esmaeili, P, Ahmadi, S, Paul, S, Wullt, B, Putze, J, Chen, SL, Dobrindt, U, Svanborg, C. Active bacterial modification of the host environment through RNA polymerase II inhibition. J Clin Invest. 2021;131(4):e140333; DOI:10.1172/JCI140333.
3. Lutay, N, Ambite, I, Hernandez, JG, Rydstrom, G, Ragnarsdottir, B, Puthia, M, Nadeem, A, Zhang, JY, Storm, P, Dobrindt, U, Wullt, B, Svanborg, C. Bacterial control of host gene expression through RNA polymerase II. J Clin Invest. 2013;123(6):2366-79; DOI:10.1172/Jci66451.
4. Sato, M, Suemori, H, Hata, N, Asagiri, M, Ogasawara, K, Nakao, K, Nakaya, T, Katsuki, M, Noguchi, S, Tanaka, N, Taniguchi, T. Distinct and essential roles of transcription factors IRF-3 and IRF-7 in response to viruses for IFN-alpha/beta gene induction. Immunity. 2000;13(4):539-48; DOI:10.1016/s1074-7613(00)00053-4.
5. Hagberg, L, Jodal, U, Korhonen, TK, Lidin-Janson, G, Lindberg, U, Svanborg Eden, C. Adhesion, hemagglutination, and virulence of Escherichia coli causing urinary tract infections. Infect Immun. 1981;31(2):564-70; DOI:10.1128/iai.31.2.564-570.1981.
6. Mobley, HL, Green, DM, Trifillis, AL, Johnson, DE, Chippendale, GR, Lockatell, CV, Jones, BD, Warren, JW. Pyelonephritogenic Escherichia coli and killing of cultured human renal proximal tubular epithelial cells: role of hemolysin in some strains. Infect Immun. 1990;58(5):1281-9; DOI:10.1128/iai.58.5.1281-1289.1990.
7. Ambite, I, Tran, TH, Butler, DSC, Cavalera, M, Wan, MLY, Ahmadi, S, Svanborg, C. Therapeutic Effects of IL-1RA against Acute Bacterial Infections, including Antibiotic-Resistant Strains. Pathogens. 2024;13(1):42; DOI:10.3390/pathogens13010042.
